# A server-assisted secure blockchain model for residential demand response in smart grids

**DOI:** 10.1038/s41598-025-31668-w

**Published:** 2026-03-19

**Authors:** Aritra Ghosh, Arup Kumar Goswami, Hassan Abdurrahman Shuaibu, Taha Selim Ustun

**Affiliations:** 1 National Institute of Technology, Silchar, Assam India; 2 Department of Electrical, Telecommunications and Computer Engineering, Kampala International University, Kampala, Uganda; 3 Fukushima Renewable Energy Institute, AIST (FREA), Koriyama, 9630298 Japan

**Keywords:** Blockchain, P2P trading, AES 256, Demand side management, Peak to average ratio (PAR), Secure server RSA, SHA 256, Green energy reward (GER), Proof of work (PoW), Energy science and technology, Engineering, Mathematics and computing

## Abstract

The paper proposes a two layered blockchain-based residential demand side management (DSM) scheme that integrates a secure centralized server, (Energy Plus) EnPlus, and a private Ethereum blockchain to deliver both real-time operational efficiency and distributed transaction trust. The architecture also allows secure and transparent peer-to-peer energy trading using (Green Energy Reward) GER Tokens, which are controlled through Solidity-based smart contracts. Computationally demanding tasks of load forecasting, bid-offer matching and policy enforcement are carried out in the EnPlus server. The authorization of prosumers is provided through Public Key Infrastructure where AES-256 encryption is used when data from smart meter collected and uploaded to server and SHA-256 hashing with RSA is used to provide data confidentiality and integrity. The shiftable appliances are scheduled through Firefly Optimization Algorithm in two conditions: DSM without P2P trading and DSM with P2P trading using tokens to reduce the energy costs. The validity of the system is brought out with a case study of 52 prosumers (25 real prosumers and 27 are part of the extended project) within the climatic conditions of Kolkata with each having a 2.5 kW rooftop photovoltaic system. This system offers better savings and reduces peak to average ratio. P2P trading has typical financial advantages and it will lead to local energy independence but there will be little delays in transactions because of the hybrid processing model. The protocol is proven to be highly scalable, privacy preserving, and robust that offers a viable roadmap to secure, decentralized residential energy markets.

## Introduction

 A paradigm shift in power grid is recorded in the modern world where information and communication technologies (ICTs) are integrated into the power generation, transmission, and distribution sectors to afford the smart grid configuration^1^. The move is driven by the proliferation of Distributed Energy Resources (DERs); rooftop photovoltaic (PV) panels, residential battery-storage, small-scale wind turbines and electric vehicles (EVs), which gradually become underlying components of residential systems^2^. These installations open opportunities to consumers to not only use grid supplied electricity but also to produce energy which can be sent back into the grid.

This has led to the transformation of conventional passive consumers to be active market players called prosumers. Prosumers, however, are involved in two-way energy transfer by selling extra production at peak periods (e.g. sunny afternoons) and purchasing energy at times of shortage or non-generation (e.g. night time)^3^. The new energy behaviour means both opportunities and challenges: the increased amount of electricity being generated and consumed locally helps to alleviate transmission losses and congestion within the grid^4^; however, fluctuations and unpredictability make it more difficult to deal with the grid^5^.

In this newly redesigned energy environment, Demand Side Management (DSM) becomes a leading force as a way to maintain grid stability. DSM programs are intended to cause prosumers to change their energy demand in response to supply situations, price signals, or grid demands. There are three main approaches contained in DSM programs: load shifting, peak shaving and demand response or DR programs. Smart grids can help to reduce carbon emissions and are supposed to improve operational efficiency, minimize energy costs, raise reliability and resilience through integrating DERs and DSM approaches^6^.

Now distributed energy resources (DER) are being introduced at the residential level at an increasing rate. For this reason, the incorporation of a broad set of dispersed prosumer market in terms of technical and organizational capabilities is particularly problematic rather than the conventional centralized control paradigm. Scalability limitations, susceptibility to privacy attacks and inherent single-point failures vulnerability are top of the list of these barriers^7^. As a result, the smart grid system of the future will have to depend on novel and decentralized coordination strategies, which generates a driving force of blockchain-based peer-to-peer (P2P) trading systems discussed later in this section.

In the residential sector, Demand Side Management (DSM) is one of the crucial options of ensuring that households have optimal use of energy. DSM has the potential to alter or reduce demand during times of peak system pressure by coordinating when appliances operate to make their use coincident with the operation of renewable generators and energy storage resources and allow user participation in local energy markets to offset variable pricing and to participate in demand response (DR) and real-time pricing system demand shifting and demand reduction programs, thus avoiding instances of overload and delaying the need to construct capital-intensive infrastructure systems^8,9^.

Nevertheless, the majority of the surviving DSM architectures are centralized which means the usage data, generated by residential smart meters, are aggregated and analysed by utilities, grid operators, or aggregators and load-control instructions, or incentivization, information is sent to the customer^10,11^. These kinds of frameworks deliver efficiency in operations, because the users has to undergo through excessive restrictions in terms of privacy, security, flexibility and resilience.

Aggregation of granular household energy data is a significant privacy threat since it can reveal potentially sensitive lifestyle patterns, occupancy patterns and use information of appliances. Consumers usually have little awareness or control in the way such data is stored, disseminated or traded^12^. Centralized schemes pose further vulnerability problems: when the centre of primary controls becomes powerless or engaged in the cyberattack, the integrity of the whole block might be jeopardised. Also, the vast interconnection of thousands or even millions of devices create great communication overheads as well as computational loads which restricts scaling^13^.

There is also transparency to prices and energy settlement arrangement in centralized DSM frameworks. But in the absence of real-time feedback, obvious incentive schemes or trackable transactions, consumers find it hard to trust in markets which is solely controlled by utilities. All of these drawbacks led to constant attempts to find decentralized and trust-less solutions. These solutions are based on blockchain, smart contract and peer-to-peer (P2P) energy trading which offer a combination of privacy protection, transparency, and self-sufficiency^14–16^.

The revolutionary technology that has defined the development of residential P2P energy-trading systems is blockchain technology characterized primarily by the following fundamental features which includes decentralization, immutability, transparency and cryptographic security. Unlike conventional centralized environments, blockchain eliminates the necessity of third intermediaries since it introduces distributed records where the verification of exchanges depends on an agreement mechanism and stores them in immutable blocks^17,18^. This is a decentralized infrastructure that allows the prosumers to directly participate in the energy markets and they can offer and demand energy based on the supply and demand conditions at a local level and they are also able to maintain integrity and traceability of data.

The introduction of smart contracts protocols automatically run on the top of the blockchain networks. It has further increased blockchain potential in reducing trust in automating the residential energy markets. Smart contract integration makes it so lucid that the energy trades can be settled in real-time. The results of the research involving in^19,20^ demonstrate that how blockchain-based solutions may build transparent, auditable, and incentive-based markets where the prosumers get compensated according to their input and compliance with the sets of rules. Moreover, to facilitate micro-transactions between its participants, these decentralized architectures allow tokenization of energy as the conversions of kilowatt-hour units into electronic assets.

However, even these benefits are nothing compared to the limitations. The full decentralized blockchain-based solutions pose on the resource-constrained residential energy ecosystems. Such public blockchains as Ethereum and Bitcoin utilize various consensus mechanisms i.e. Proof of Work (PoW) and Proof of Stake (PoS), respectively. Although these methods possess high levels of security aspects and at the same time, they possess latency and capacity constraints which makes them unsuitable to the real-time requirements of a domestic demand-side management operation. In particular, the relatively slow throughput of transactions in the Ethereum system and the energy with which it is conducted can prove to be barriers to scalability and to the sustainability goals on the applications on residential scale.

Additionally, in a number of blockchain ecosystems, Identity and Access Management (IAM) frameworks are not integrated, which makes the verification of certifiably genuine prosumer device or user through a requirement of non-repudiation, maintainability, and regulatory compliance, cumbersome. Moreover, supporting regulatory control, the lack of interoperability with grid infrastructure, challenges to coordinate the motivation behind tokens and the price structure of existing markets still remain as roadblocks. These challenges are the reasons why the direct application of public blockchain models to handle mission-critical energy management processes has not been used and instead, a search is underway into hybrid or server-aided blockchain variants that can balance decentralization and performance, control, and trust. The changing environment requires the creation of customary blockchain frameworks of residential energy trading which features lightweight, robust, auditable pertaining to interoperability with smart-grid ecosystems.

The systemic constraints of any fully decentralized blockchain infrastructure, or, in other words, latency, limited scalability, energy waste, and the inability to credibly verify identity, have generated the provision of hybrid and server-assisted blockchain systems as the superior architectural solutions. These models attempt to strike a balance between distributed and centralized control by tapping the robustness and auditability of distributed ledger systems, and also simultaneously embracing the computational performance provided by the secure centralized servers.

A specific purpose of central servers/trusted nodes in this configuration is to perform resource-demanding functions, which includes demand forecasting, pricing optimization, user authentication, speed-up of consensus, secure storage of transactions, decentralized verification and smart contracts execution. Such modular organization would maintain the fundamental advantages of blockchain’s immutability, transparency, distributed control, and address inefficiencies in operations of residential DSM environment.

A striking example is offered by the SynergyGrids framework^25^, that combines a hybrid blockchain system with the centralized controller to coordinate the market in real time, predict demand and distribute tokens. The simulation of this system found more than 17% reduction in energy expenses and better matching of the demand and supply in a community of residential prosumers. This moved complex time sensitive calculations to a cloud managed controller by securing information on the ledger about bid and offer data, and resultantly created a scalable local energy market that is responsive.

Another strong architectural solution on decentralised energy markets is RETINA (REal-Time energy trading INfrAstructure)^26^. It is a permissioned blockchain-based platform combined with the Public Key Infrastructure (PKI) to facilitate secure access control, manage prosumer identity, and execute role-based authorisation. The permissioned topology of RETINA would guarantee that only recognised and authorised members can take part in the market thus preventing spoofing, Sybil attack, and unauthorised access which are often not considered in the implementation of public blockchains. PKI-enabled nodes are also blended with smart contracts which regulate the trading behaviour and grid policies which are compliance-driven, secure, and transparent energy trade.

The hybrid systems like RETINA also tie in better with smart grid regulatory regimes, which regularly require traceability of identity, metering accuracy, transparency of billing and capability of grid operators to intervene. This means that server-assisted blockchain solutions give compliance-ready, grid-friendly alternative to a conventional blockchain application. These systems are of specific importance while implementing the server-assisted blockchain in residential applications with a demand-side management because the three features mentioned (high transaction throughput, real-time processing, and data privacy) are essential in this scenario.

In this paper, a blockchain architecture supported by secure servers is developed and proposed that supports residential demand-side management (DSM) of the smart grid, and allows a secure, transparent, and efficient peer-to-peer (P2P) energy trading between prosumers. The main contributions are the following ones:

i) An original hybrid structure is presented that combines the decentralized functions of blockchain architecture and a flexible schedule and security provided by a centralized server. The architecture facilitates the security management and transaction monitoring in compliance with the regulatory requirements without violating the distributed integrity of energy trade.
ii) An Improved token-based trading system is implemented by a cryptographic token called Green Energy Reward (GER) that allows P2P trade in terms of energy between residential prosumers. Trading process is automated using smart contracts which provide fairness, traceability, and avoid ability in carrying out transactions, as well as guarantee its tamper-free execution.
iii) The prototype is tested and simulated between 52 prosumers of Rabi Abason extended project where all prosumers have the generation capabilities of 2.5 kW from their rooftop solar panel. The real time data of 25 prosumers are fetched and trained in the LSTM model for Short Term Load Forecasting. Through this also 27 extended household consumption and generation patterns are executed.
iv) Based on Generation and consumption data Demand Response program is executed using Appliances Scheduling considering Prosumer’s preferable time frame. But, in contrast to traditional Demand Side Management (DSM) models that are based purely on centralized utility-driven scheduling, the given work proposes the addition of a secure peer-to-peer blockchain layer in which the prosumers engage in direct energy surplus trading using tokenized transactions.
v) EnPlus secure server has incorporated identity management using PKI, AES-256 encryption of smart meter data, and RSA with SHA-256 digital signatures to sign blockchain level transactions. Such a hybrid solution increases confidentiality, integrity, and authentication all at once.
vi) Beyond static DSM approaches a dynamic, decentralized energy market is introduced by the incorporation of real time tokenized bidding mechanism.
vii) A full stack implementation process is introduced from smart meter data encryption to solidity based blockchain deployment with JSON encoding of transaction.

## Literature survey

Modern power system growth accompanied by increased power system flexibility with a growing number of Distributed Energy Resources (DERs) including rooftop solar panels, energy storage devices, and electric vehicles has created the need to have smarter, more decentralized, and secure methods of residential energy management. Therefore, the potential of blockchain and others to increase transparency, security and autonomy in residential demand-side management (DSM) systems has been explored by some scholars.

The groundbreaking work by Satoshi Nakamoto on Bitcoin^39^ set the stage of decentralized transaction processing, sparking off the consideration of the use of blockchain that is in no way confined to cryptocurrencies. In the energy market, the need to have secure, trust-less and auditable transactions made it a very attractive field.

Bera et al.^25^ proposed SynergyGrids that is a mix of blockchain-based architecture combining server-side forecasting and decentralized trading. In their simulation, their model reduced the energy costs by 17 per cent, which then showed that server assistance can be used to enhance decision-making and scalability. Similarly, RETINA joins Public Key Infrastructure (PKI) and permissioned blockchain to improve access management and perform user authentication to cope with the Identity-related vulnerabilities inherent with decentralized networks.

Morstyn et al.^27^ focused on the design of the market of decentralized flexibility services and offered algorithms of trading that allowed prosumers to participate in balancing the demand. Wang et al.^28^ followed this paper, by addressing the similar problem using a game-theoretic approach to energy trading, emphasizing the role of incentives and modelling of behaviour in peer-to-peer (P2P) grids.

To provide a detailed overview of the blockchain energy environment, Andoni et al.^29^ categorized the use-cases and defined technical and regulatory challenges. That is, their results coincide with those of Pilz and Al-Fagih^30^ who have suggested an incentive-compatible DSM model based on smart contracts, which has been focused on privacy and fairness.

The current review is an analysis of the landscape and scenery of blockchain-empowered distributed energy management (DEM) systems, especially about scalability and transaction security. Two studies providing evidence on smart-contract-based architectures of energy microgrids and energy trading platforms by Goranovic et al.^31^ and by Yang et al.^32^ are reported. Any consistent results in the two works deduce that blockchain technology can be used to process transactions safely and automatically, but shows the disadvantage of performance and integration issues when it has been applied to real-time demand-side management (DSM).

Park et al.^33^ carried the vision of smart cities by presenting the idea of blockchain that can support sustainable urban energy. Aitzhan and Svetinovic^21^ proposed a decentralized energy trading platform where multi-signature schemes and anonymized data-flow were used in an attempt to enhance the security and privacy. Liang et al.^34^ introduced ProvChain, a privacy-enhanced provenance system that may be customized to that of DSM where non-repudiation and traceability are of serious concern.

Xu et al.^36^ presented an IoT architecture using blockchain believing that it has applicability in energy applications where edge IoT devices have to be under secure control.

Together these works pointed out a number of emerging themes: hybridization of architectures, where both centralized forecasting and control are combined with decentralized transactions; the sensitivity of the system to security practices like PKI and multi-signature schemes; viability of incentive-based trading mechanisms; and scalability limits of current public blockchain systems.

Despite such advances, there still exists significant holes (mentioned in Table 1) in the aspects of real-time energy trading, secure identity management and scalable architecture. The current contribution addresses these gaps as it introduces a server-assisted blockchain model that would ensure peer-to-peer energy trading with strong authentication, effective smart contract logic, and verifiable transaction ledgers.

**Table 1:** Research gap identification and establish the paper contribution.

Ref. No.	Hybrid Blockchain Model	Scalability of Public Blockchain	Privacy & Identity	Token Governance	Real-time DSM	Realistic Implementation
^25^	✔	✔	✔		✔	✔
^27^		✔				
^28^		✔				
^29^			✔			
^30^				✔		
^31^		✔		✔		
^32^					✔	✔
^33^				✔	✔	
^21^			✔			
^34^			✔			
^35^		✔		✔	✔	
^36^		✔	✔			✔
^37^						✔
^38^	✔	✔	✔			✔
This Paper	✔	✔	✔	✔	✔	✔

## System architecture and methodology

### Overview

In the present research work, a hybrid blockchain framework has been proposed that is aimed at providing secure, scalable, and efficient Demand Side Management (DSM) solutions in the residential spheres. The architecture combines decentralized and centralized paradigm, hence linking the transparency and finality that come with the use of blockchain technology with the efficiency of computations and the capability to control a secure server, what is known as EnPlus. Such a layered architecture allows ensuring secure peer-to-peer (P2P) energy trading between residential prosumers, at the same time guaranteeing real-time data processing, trustless token exchange, and verifiable smart contract execution. The architecture is made of the three following integrated components:

#### Prosumer interface layer

Every household prosumer is equipped with a Smart Energy Management System (SEMS) that is directly connected with a smart meter and the EnPlus server. SEMS is a system that allows monitoring real-time energy generation, consumption, and market participation. It serves as the initial contact of bidding, offering, and receiving tokens and manages safe messages with EnPlus server through encrypted routes.

#### EnPlus secure server layer

The main objective of the proposed hybrid blockchain-secure-server architecture is to combine decentralized trust with centralized computational efficiency for residential DSM. The EnPlus server functions as the *computational backbone.*The centralized EnPlus server does the authentication with Public Key Infrastructure (PKI) and does the computationally demanding tasks like load forecasting, bid-offer matching and DSM optimization. It also plays the role of decision-making authority that carries out operational policies, token constraints and sieves out invalid transactions before relaying them on to the blockchain to be recorded.

#### Blockchain network layer

Smart contract deployment, the validation of token exchanges and maintenance of ledgers are conducted within a private, permissioned Ethereum-based blockchain network. After a validated trade has been accepted by the EnPlus server, the associated smart contract is then run and recorded permanently on the block chain. This design provides integrity, auditability and resistance to tampering of data.

The hybrid model therefore offers the balance between the decentralization of trust and centralization of processing allowing the large number of prosumers to be accommodated and maintain the system responsiveness and compliance with the regulatory framework.

**Fig. 1:**
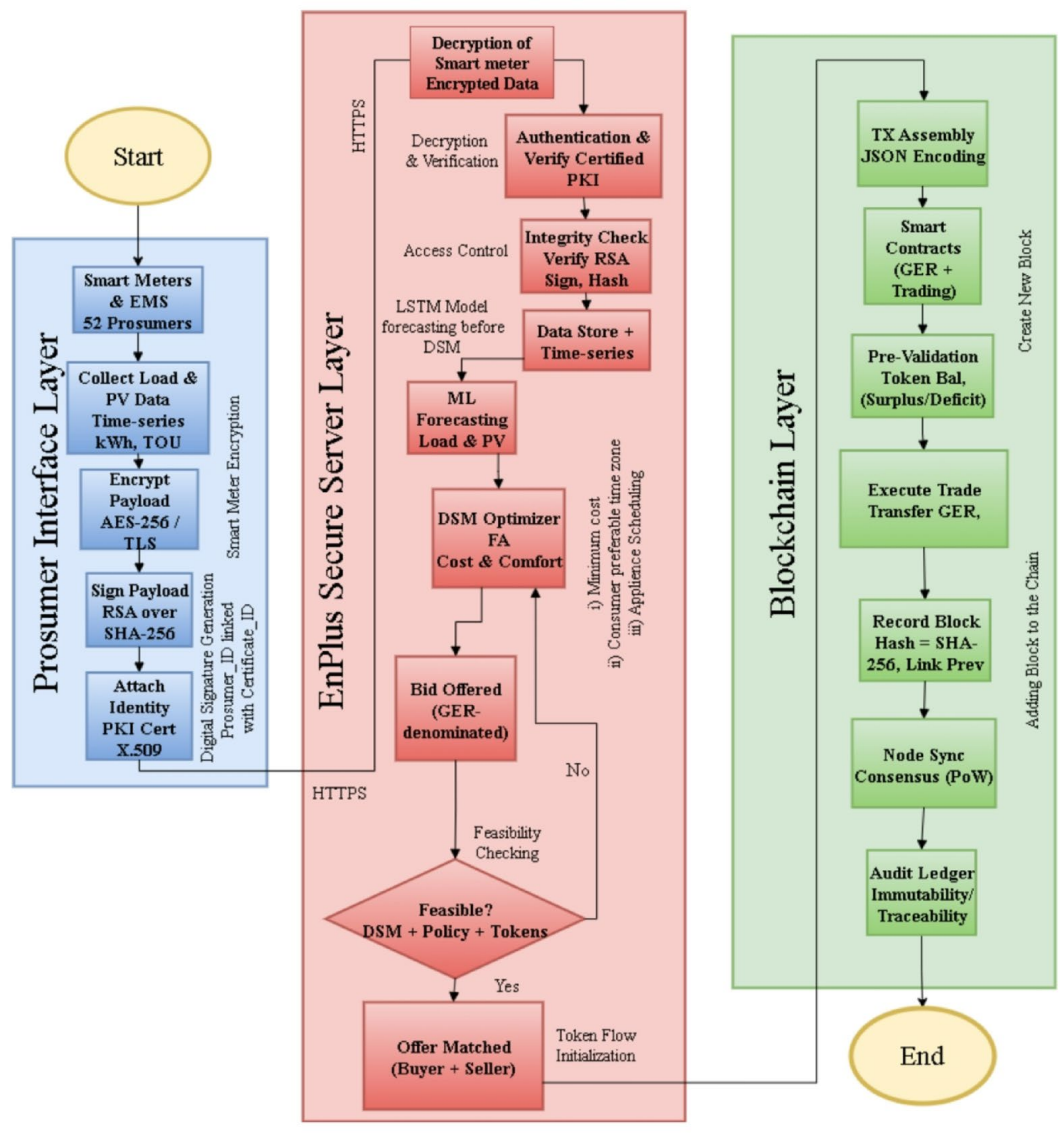
Overall architecture.

The Fig. 1 characterizes the working model of the suggested hybrid blockchain architecture of residential demand-side management. The workflow starts with prosumers who in this case are the 52 households that send encrypted energy-trade offers to the centralized EnPlus server via the Smart Energy Management Systems (SEMS). Before processing the bid, EnPlus server authenticates every prosumer with Public Key Infrastructure (PKI), applies machine-learning demand forecasts, and conducts bid-offer matching with respect to current load profiles and DSM constraints.

When the validation is successful, the subsequent transactions are coded in the form of smart contracts and posted on the blockchain ledger, which protects transparency and immutability. Prosumers are then sent token settlement messages and the ledger is then open to regulatory audit and compliance monitoring. On the other hand, all the transactions, which are not valid or are fraudulent, get eliminated before the blockchain is implemented, thus maintaining system integrity and performance.

Overall, the flowchart combines the real-time decision-making with the distributed trust mechanism, and provides a solid basis of secure p2p energy trading.

### Smart meter data collection and encrypted upload to enplus secure server

**Fig. 2:**
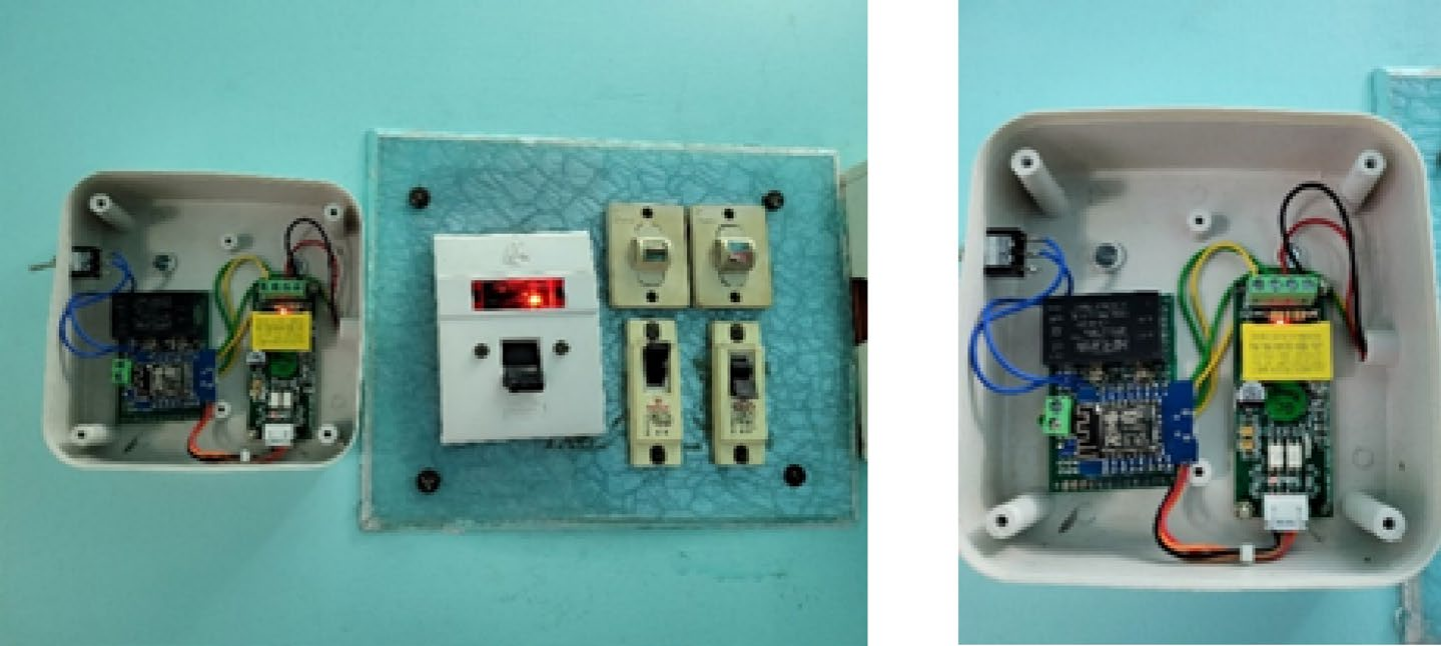
ESP32 based smart meter.

The process is initiated by the installation of ESP 32 based smart meters in Fig. 2 at the premises of each prosumer that constantly collect granular data on energy consumption and real-time prices of electricity calculated on dynamic time-of-use (ToU) tariffs (attributes are mentioned in Table 2). Every prosumer is distinctly identified by a cryptographically generated *Prosumer_ID* which is linked to a *Certificate_ID* by the Public Key Infrastructure (PKI) and is thus authentically identified and capable of committing to the system securely. After the consumption and pricing data are aggregated, smart meter encrypts it with AES-256 encryption algorithm and sends securely via HTTPS.This encryption will ensure the confidentiality and integrity of the data. The encrypted data is decrypted after arriving to the ENPlus secure server with the help of the respective decryption key, checked to be authentic, and finally stored securely. Authentication-based access control is also possible on this layer, which will guarantee that only authorized parties will be able to retrieve or update the data. HTTPS ensures a secure transmission channel and digital certificates ensure authentication of access; thus, the framework provides a secure environment to acquire data in real-time and make decisions as well as an environment to trade energy in a decentralized framework.

**Table 2:** Data coming from smart meter.

Attributes	Description
T (Timestamp)	Date and time of reading
E_C_ (Energy Consumed)	kWh consumed during interval
E_G_ (Energy Generated)	kWh generated via solar
P (Price per kWh)	Time-of-Use electricity price
*Prosumer_ID*	Unique ID of the prosumer

#### Data encryption

**Fig. 3:**
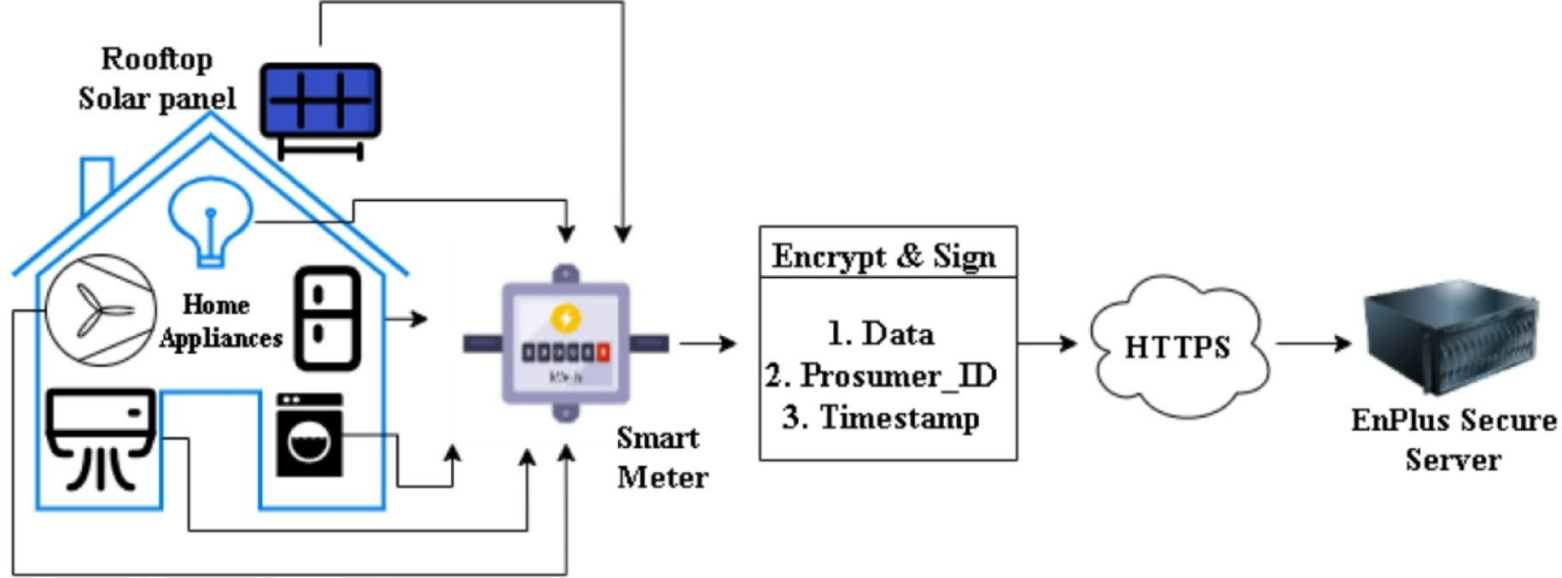
Data collected and uploaded to EnPlus secure server.

For the Data encryption purpose AES 256 is implemented here. Now AES 256 is a Block cipher that encrypts 128-bit plain text block ($\:P\epsilon\:{\mathbb{Q}}_{128}\mathrm{)}$ to 128-bit cipher text block ($\:C\epsilon\:{\mathbb{Q}}_{128}\mathrm{)}$ by using 256-bit symmetric key ($\:K\epsilon\:{\mathbb{Q}}_{256}\mathrm{)}$. Where, $\:\mathbb{Q}$ denotes the finite field of binary element. *P* and *C* are the plaintext and ciphertext respectively and *K* is 256-bit key. The process is explained in Fig. 3.

Now, here from key schedule function Round Key (R_i_) will be generated for each round from *K.*

Here, $\:{R}_{i}\epsilon\left\{{R}_{0},{R}_{1}\:,{R}_{2},{R}_{3}\dots\:\dots\:\dots\:.{R}_{14}\right\},$ where in AES256 algorithm 14rounds are used.

For initial round (i = 0)

1 $$\:{S}_{i}=P\oplus\:{R}_{i}\:$$

For Intermediate round (i = 1 to 13)

2 $$\:{S}_{i}=M\left(\sigma\:\left(S\left({S}_{i-1}\right)\right)\right)\oplus\:{R}_{i}$$

For final round (i = 14)

3 $$\:C=\sigma\:\left(S\left({S}_{i-1}\right)\right)\oplus\:{R}_{i}$$

Where $\:M\left(\right)$ and $\:\sigma\:\left(\right)$ are two functions used to interchange column and rows and $\:{S}_{i}$ is the sub byte transformation.

After reaching to the EnPlus Server this encrypted data is decrypted. For decryption procedure $\:{\sigma\:}^{-1},\:{M}^{-1}$ and $\:{S}^{-1}\:$inverse is calculated in the EnPlus Server.

### Load forecasting

Effective demand-side management (DSM) of residential energy systems depends on a proper evaluation of electrical load. In the proposed structure, a combined load-forecasting system is incorporated in the EnPlus secure server to enable decision-making when it comes to energy trading, bid matching, and scheduling. The forecasting process utilizes the machine-learning models that are trained on the past consumption and generation data retrieved in the Rabi Abason smart housing complex.

#### System description

Rabi Abason currently has twenty-five prosumer households, each with a 2.5-kW rooftop solar photovoltaic (PV) system. Every house has a smart energy meter, which stores data about electricity consumed and PV generated every 15 min. The data used in this paper covers 90 days of consecutive data where parameters like solar irradiance, temperature, load consumption and PV output were measured at their corresponding time resolution.

The system-expansion plan requires that the infrastructure of the system be able to accommodate an increment of twenty-seven prosumers, which makes it necessary to have a forecasting framework that can be accurate and scalable to the incremental data volume.

#### Machine-learning approach

In short-term load forecasting (STLF), a Long Short-Term Memory (LSTM) neural network has been embraced because it has proven to be able to incorporate temporal dependencies in time-series data. The model is trained to forecast the next 24 h load pattern based on the previous seven days of hourly data on consumption and PV generation.

The basic Load Balance is explained in Eq. (4)

4 $$\:{D}_{i}\left(t\right)={E}_{Ci}\left(t\right)-{E}_{Gi}\left(t\right)$$

Where $\:{D}_{i}\left(t\right),{E}_{Ci}\left(t\right),{E}_{Gi}\left(t\right)$ are the net power demand, Energy consumption and Energy Generated (solar power) at time t.

Now LSTM model forecasted the future net demand explained in Eq. (5)

5 $$\:\widehat{{D}_{i}}(t+1)=\:{f}_{LSTM}\left({D}_{i}\left(t\right),{D}_{i}\left(t-1\right),{D}_{i}\left(t-2\right)\dots\:\dots\:..{D}_{i}\left(t-n\right)\right)$$

Where $\:\widehat{{D}_{i}}(t+1)$ is the forecasted net demand for time $\:(t+1)$.

$\:{f}_{LSTM}$ is the function of LSTM model trained per prosumer.

This model is trained to minimize the mean square explain in Eq. (6).

6 $$\:{MSE}_{i}=\frac{1}{n}\sum\nolimits_{t=1}^{n}{\left(\widehat{{D}_{i}}\left(t\right)-{D}_{i}\left(t\right)\right)}^{2}$$

Also, coefficient of Determination (R^2^) is expressed in Eq. (7).

7 $$\:{{R}^{2}}_{i}=1-\frac{\frac{1}{n}\sum\nolimits_{t=1}^{n}{\left(\widehat{{D}_{i}}\left(t\right)-{D}_{i}\left(t\right)\right)}^{2}}{\frac{1}{n}\sum\nolimits_{t=1}^{n}{\left(\stackrel{-}{{D}_{i}\left(t\right)}-{D}_{i}\left(t\right)\right)}^{2}}$$

At the beginning the dataset of 25 prosumers at Rabi Abason was collected using ESP-32 smart meters. Now this community had a plan to extend the number of solar houses to 52. To work on this extended plan 27 more houses data should be incorporated in the En-Plus blockchain framework. Now to evaluate the extended scenario 27 load profiles were generated statistically using LSTM model with the same distribution pattern where mean, variance and diurnal pattern are the key factors.

The extension plan had been executed using the transfer learning model of LSTM.

Under the extension plan to calculate the load pattern of non-existed prosumer 26, first $\:{MSE}_{26}$is calculated using transfer learning.

8 $$\:{MSE}_{26}=\:\overline{{\mu}_{MSE}}+{{\Delta}}_{MSE}$$

where $\:{{\Delta\:}}_{MSE}={\lambda\:}_{TL}\stackrel{-}{{\mu\:}_{MSE}}$, here $\:{\lambda\:}_{TL}$is the transfer learning loss factor.

Similarly, $\:{R}^{2}$ is decrease due to the increment of MSE.

9 $$\:{R}_{26}^{2}=\overline{{\mu\:}_{{R}^{2}}}-{\Delta\:}{R}^{2}\:\mathrm{w}\mathrm{h}\mathrm{e}\mathrm{r}\mathrm{e}\:{\Delta\:}{R}^{2}={\lambda\:}_{TL}\overline{{\mu\:}_{{R}^{2}}}$$

Now depending on the Load Pattern and PV utilization all 25 prosumers are distributed into 3 clusters.

10 $$\:\left[\begin{array}{c}{C}_{1}=\left\{2,\:5,\:7,\:9,\:14,\:17,\:20,\:25\right\}\\\:{C}_{2}=\left\{1,\:3,\:6,\:10,\:12,\:16,\:18,\:23\right\}\\\:{C}_{3}=\left\{4,\:8,\:11,\:13,\:15,\:19,\:21,\:22,\:24\right\}\end{array}\right]$$

As beyond 25 the prosumers fall under the extension plan. So, no real data is available from the prosumer no 26 to 52. Hence the Euclidean based approach of selecting cluster is not applicable here. So, the prosumers (26–52) are assigned in the cluster on random basis.

11 $$\:\left[\begin{array}{c}{C}_{1}=\left\{26,\:29,\:31,\:35,\:38,\:44,\:48,\:52\right\}\\\:{C}_{2}=\left\{27,\:30,\:33,\:36,\:40,\:43,\:46,\:49\right\}\\\:{C}_{3}=\left\{28,\:32,\:34,\:37,\:39,\:41,\:42,\:45,\:47,\:50,\:51\right\}\end{array}\right]$$

Now forecasted load in non-existing prosumer from (26–52) is

12 $$\:\widehat{{D}_{i}}\left(t\right)=\overline{{D}_{i}^{k}}\left(t\right)+{\epsilon}_{t}$$

where $\:{\epsilon}_{t}=\mathcal{N}\left(0,{\sigma\:}_{MSE}\right)$ represents the error and variance $\:{\sigma\:}_{MSE}=\:\sqrt{MSE}$.

$\:k$ is total no of cluster and $\:\stackrel{-}{{D}_{i}^{k}\left(t\right)}$ represents the cluster mean. Where $\:{C}_{k}=\left\{\mathrm{1,2},\dots\:..25\right\}$.

### Demand side management in enplus server

**Fig. 4:**
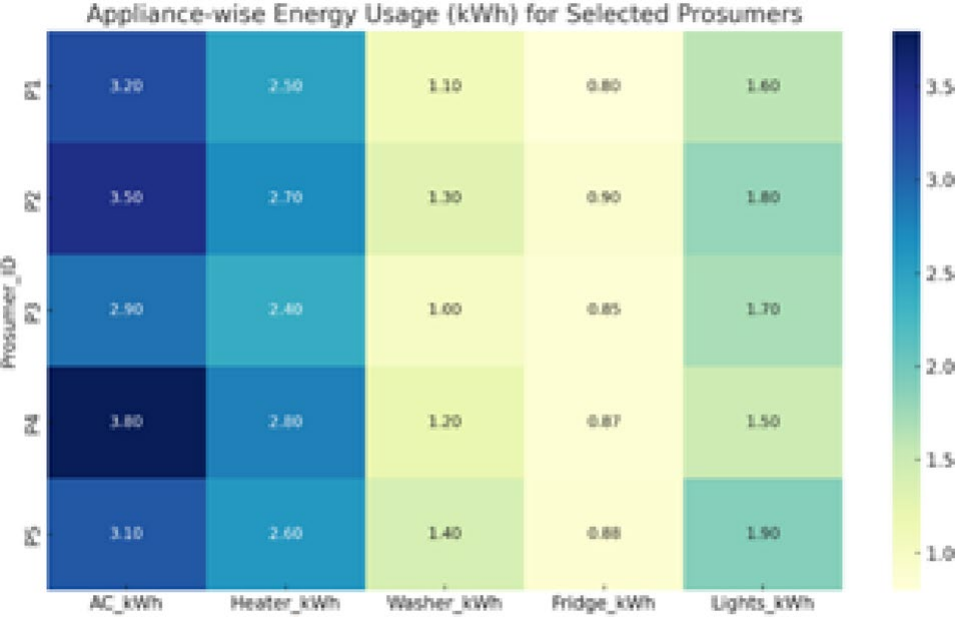
Heat map of 5 prosumers with 5 main consumable and shiftable appliances.

Residential demand side management plays a vital role for energy conservation, reduction in peak load and improve peak to average ratio (PAR). This is actually beneficial for the utility grid to running on low burden as well as the consumer end to enjoy the profitability. In this case consumers are also generating the electricity through their rooftop mounted solar panel. First one is the centralized approach where DR program is written in the EnPlus server, based on that program, ToU tariff and consumer preference are taken into consideration to shift the load from on peak hour to off peak hours. In this traditional approach the solar power is considered in forecasting and explained in Figs. 3 and 4. But in the next case in decentralized approach peer to peer trading between prosumers are taken into consideration. Here excess solar generation either utilised by the prosumer itself or is updated for bidding in the peer-to-peer network.

#### Centralized approach

A centralized DSM policy tries to ensure that the total cost of grid electricity is reduced and the satisfaction of the prosumers is raised. The prosumer satisfaction is a combination of the profitability and the time of usage of the appliances. Now the basic appliances which need to be shifted are in the following Fig. 4.

***Mathematical Foundation***

The basic objective function is to minimize the total cost of energy consumption mentioned in Eq. (13).

13 $$\:C=min\sum\:_{i=1}^{N}\sum\nolimits_{a\epsilon{A}_{i}}\sum\nolimits_{t=1}^{T}\lambda\:\left(t\right){P}_{i,a}\left(t\right){x}_{i,a}\left(t\right)-\alpha\:\sum\nolimits_{i=1}^{N}\sum\nolimits_{a\epsilon{A}_{i}}\sum\nolimits_{t=1}^{T}{\omega\:}_{i,a}\left(t\right){x}_{i,a}\left(t\right)$$

Where, $\:N$ is the number of prosumers, $\:{A}_{i}$ is the set of appliances for prosumer $\:i$ and $\:T$ is the total number of time slots i.e. 24 h. Now, $\:{P}_{i,a}\left(t\right)$ is the power consumption of appliance and $\:\lambda\:\left(t\right)$ is the Time-of-use (TOU) price at time slot $\:t$. Satisfaction Weighting Factor $\:\alpha\:$ for balancing cost reduction and maintaining comfort is introduced in the Eq. (8). $\:{x}_{i,a}\left(t\right)$ and $\:{\omega\:}_{i,a}\left(t\right)$ are two factors described in Eq. (13a) and (13b).

$\:{x}_{i,a}\left(t\right)\in\:\left\{\mathrm{1,0}\right\}$ = Binary decision variable (1 if appliance a is ON at time $\:t$, 0 otherwise) (13a).

$\:{\omega\:}_{i,a}\left(t\right)\in\:\left\{\mathrm{1,0}\right\}$= Satisfaction weight of appliance $\:a$ at time $\:t$ (13b).

Now at the time of the execution of the Demand Response Program three constraints are taken into consideration. These are appliance energy requirement, Operation time window frame and Prosumer satisfaction, which are mentioned in Eqs. (14)-(16).

14 $$\:\sum\nolimits_{t=1}^{T}{P}_{i,a}\left(t\right){x}_{i,a}\left(t\right)\varDelta\:\mathrm{t}={E}_{i,a}\:\mathrm{w}\mathrm{h}\mathrm{e}\mathrm{r}\mathrm{e}\:\forall\:i,\forall\:a\in\:{A}_{i}$$

15 $$\:{x}_{i,a}\left(t\right)=0\:\mathrm{i}\mathrm{f}\:\:\:\:\mathrm{t}\notin\:\left[{t}_{i,a}^{max},{t}_{i,a}^{min}\:\right]$$

16 $$\:\mathcal{K}=\sum\nolimits_{t=1}^{T}{\omega\:}_{i,a}\left(t\right){x}_{i,a}\left(t\right)$$

Where $\:\mathcal{K}$ is the Prosumer satisfaction index and it becomes high when prosumer preferable time is matched with the appliance operating time.

#### Decentralized approach

In this process the optimization is based on two cost parameters-i) Energy purchased from the grid and ii) Energy exchange via token.

***Mathematical Foundation***

Now this is written as follows in Eq. (17).

17 $$\:C=min\sum\nolimits_{i}^{N}\sum\nolimits_{t}^{T}\left[r\left(t\right){E}_{i}^{Grid}\left(t\right)+{\gamma\:}_{t}\left(\sum\nolimits_{j=1,j\ne\:i}^{N}{\tau\:}_{i,j}\left(t\right)-\sum\nolimits_{j=1,j\ne\:i}^{N}{\tau\:}_{j,i}\left(t\right)\right)\right]$$

Now the energy balance equation is written in Eq. (18)

18 $$\:{E}_{G,i}\left(t\right)+\:{E}_{i}^{Grid}\left(t\right)+\:{E}_{i}^{buy}\left(t\right)=\:{E}_{c,i}\left(t\right)+{E}_{i}^{Sell}\left(t\right),\:\:\:\:\forall\:i,t$$

Where, $\:r\left(t\right)$ is the grid electricity price at time $\:t$ based on the TOU tariff. $\:{E}_{i}^{buy}\left(t\right)$ and $\:{E}_{i}^{Sell}\left(t\right)$ are the energy purchase or sell by the $\:ith$ prosumer at time $\:t$. $\:{\tau\:}_{i,j}\left(t\right)$ and $\:{\tau\:}_{j,i}\left(t\right)$ are the token exchange from $\:ith$ prosumer to $\:jth$ prosumer and vice-versa.

 The token exchange policies are explained in Sect. “Token-based Peer-to-Peer (P2P) energy trading mechanism”.

## Token-based Peer-to-Peer (P2P) energy trading mechanism

The tokenized trading model, which is the basis of the proposed system, allows ensuring the safety, decentralization, and traceability of the prosumer-to-prosumer energy exchange. A digital token, the GER (Green Energy Reward) is proposed in this paper to quantify a measurable unit of energy transfer through smart contracts. The arrangement eliminates the use of a central clearinghouse, and therefore enhances fairness, transparency and tamper resistance in transaction settlement.

### Mathematical modelling of token exchange

First a set of participating prosumers is taken into consideration.

19 $$\:P=\{{p}_{1},{p}_{2},{p}_{3},{p}_{4},{p}_{5},{p}_{6},{p}_{7}\dots\:\dots\:..{p}_{N}\} \: \mathrm{where}\: N=52=\text{Total No of Prosumers}.$$

Now token generation and exchange rule is given by

20 $$\:{\tau\:}_{i,j}\left(t\right)=\frac{{E}_{i,j}\left(t\right)}{\partial\:}\:$$

where $\:{\tau\:}_{i,j}\left(t\right)$ is the token transferred from $\:{p}_{i}$ to $\:{p}_{j}$ at time $\:t$. Similarly, $\:{E}_{i,j}\left(t\right)$ is the amount of Energy transferred from $\:{p}_{i}$ to $\:{p}_{j}$ at time $\:t$. $\:\partial\:$ is denoted as the conversion rate (kWh/Token).

After each transaction the token balance of $\:{p}_{i}$ is updated according to Eq. (21).

21 $$\:{\tau\:}_{i}\left(t+1\right)={\tau\:}_{i}\left(t\right)+\:{\tau\:}_{i,j}\left(t\right)$$

Similarly, $\:{p}_{j}$ is updated in Eq. (22).

22 $$\:{\tau\:}_{j}\left(t+1\right)={\tau\:}_{j}\left(t\right)-\:{\tau\:}_{i,j}\left(t\right)$$

But Eq. (11) to (14) can be implemented considering the constraints in Eqs. (23) and (24).

23 $$\:{E}_{i,j}\left(t\right)\le\:{E}_{i}^{surplus}\left(t\right)$$

24 $$\:{\tau\:}_{j}\left(t\right)\ge\:{\tau\:}_{i,j}\left(t\right)$$

Equation (15) and Eq. (16) are energy availability and token affordability constraints respectively.

Now smart contract rule is fixed and updated according to Eq. (25)

25 $$\:IF\:\left({E}_{i,j}\left(t\right),{\tau\:}_{i,j}\left(t\right)\right)\:is\:valid==\:Transaction\:Executed$$

### Trading process

**Fig. 5:**
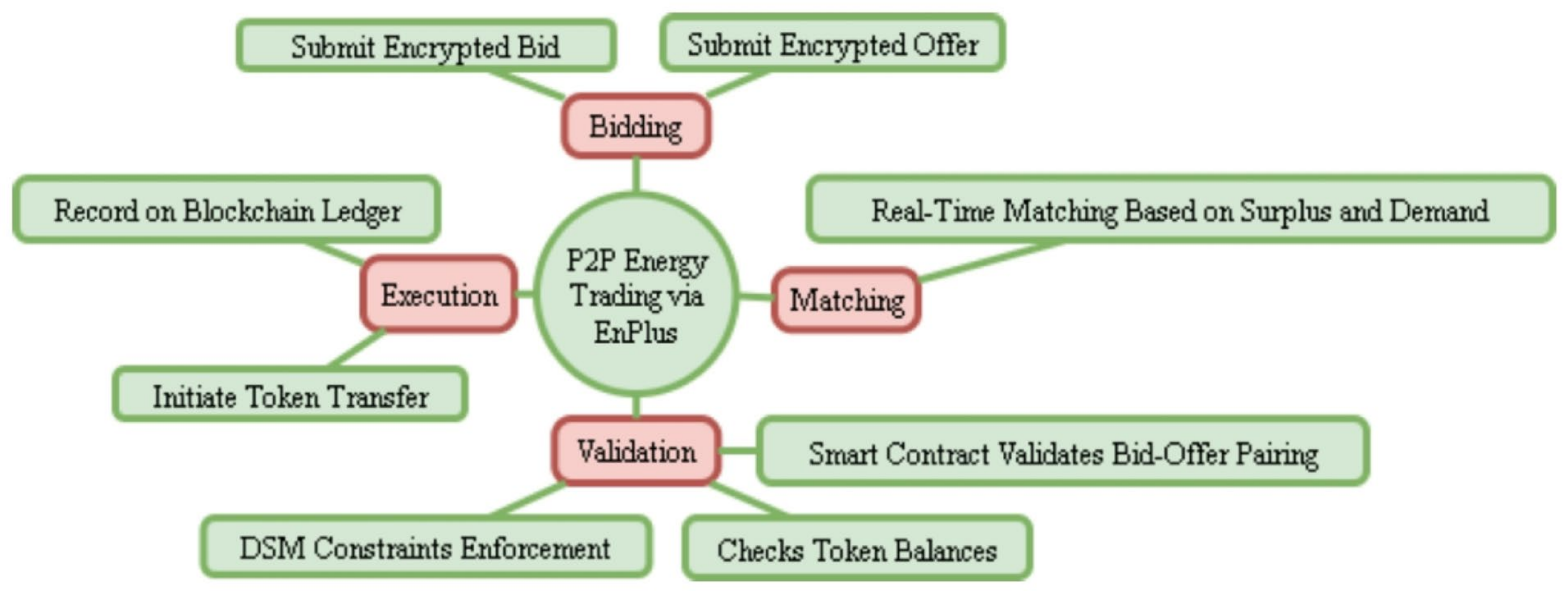
Overall trading process.

In peer-to-peer energy trading, EnPlus platform starts its work with the bidding stage where residential prosumers can place encrypted energy bids and offers in order to protect privacy. In the matching stage, the EnPlus server uses current energy surplus and demand records and matches buyers to appropriate sellers in real time. When a match is found, the so-called validation phase is performed; in this phase, a smart contract ensures that transactions are valid by ensuring three conditions; the bid-offer pairs, there are enough tokens available, and the demand-side management (DSM) constraints are satisfied by load scheduling and energy availability. After the successful validation, the execution phase is activated, which automatically transfers the tokens between prosumers, and the whole process is stored in the blockchain ledger, which eliminates the loss of transparency, traceability, and trust explain clearly in Fig. 5.

In the architecture of the peer-to-peer (P2P) energy trading powered by blockchain, the use of crypto allows an efficient and secure distribution of excess energy among the prosumers. Every unit of energy that circulates is represented by a cryptographic token, which in this case will be referred to as Green Energy Reward (GER) token. Prosumers with surplus energy produced by rooftop solar panels or other distributed energy resources can sell that surplus to prosumers in their neighbourhood by exchanging GER tokens. On the other hand, tokens are used by the buyers to obtain energy, thus allowing open, traceable, and safe payments on the blockchain ledger.

### Bid matching

**Fig. 6:**
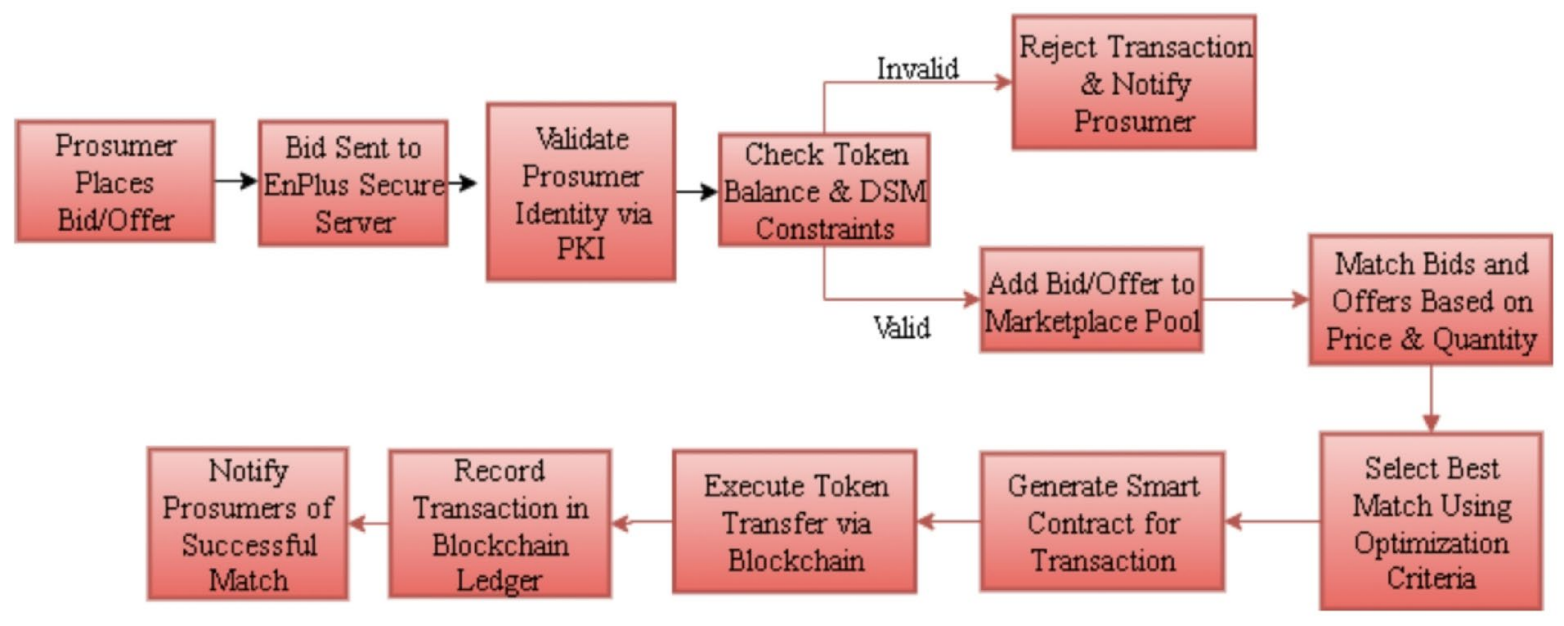
Bid matching process.

Bid matching is the mechanism by which an energy excess created by a seller can be matched with an energy deficit manifested by a buyer provided that it is done in a coordinated way with respect to price, quantity and time. Under the current architecture, the prosumers will bid and offer through the EnPlus interface, after which encrypted information (AES-256 with public-key infrastructure) is transferred to the EnPlus server. The server uses pre-set matching rules and when they are satisfied, the server initiates the running of a smart contract to be followed by settlement. Now flowchart in Fig. 6 will explain the procedure of bid matching and Table 3 indicates the platform used in different stages.

**Table 3:** Different process implementation platform.

Function	Platform	Purpose
Smart-meter simulation	ESP-32 C firmware	Data acquisition
Forecasting	Python	Load prediction
Optimization	Python based Firefly Algorithm	Appliance scheduling
Blockchain	Ethereum Remix IDE + Ganache + MetaMask	Smart-contract deployment
Server	Python EnPlus API	Real-time data handling

## Data accessibility and security using blockchain

### Overview

In a decentralised energy-trading ecosystem, a security and verification is needed to create digital identities of all participating prosumers, that can lead to develop a system which is trustworthy, traceable and perform transactional integrity. In order to fulfil this need, Blockchain uses Public Key Infrastructure (PKI) to formulate the principle of identity management, authentication and secure communication. Every prosumer has its distinct digital identity which is depicted as a key pair, a public key ($\:{\kappa\:}_{pub}$) and a private key ($\:{\kappa\:}_{pvt}$). They are created with the help of asymmetric cryptographic RSA algorithms which makes it possible to encrypt, sign and verify information. Public key is sharable within the blockchain but private key must belong to the prosumer. Every authenticated information exchange between the prosumer and the smart contract or the prosumer and the EnPlus Server should be digitally signed by the private key. Public key is there for the authentication purposes. A trusted Certificate Authority (CA) is used to issue a Digital Certificate that binds the prosumer public key to Prosumer_ID. It Is mandatory for the certificate to comply with the X.509 standard and it will include metadata that will consist of the certificate serial number, certificate issuer, certificate validity, and the smart meter device ID. EnPlus ecosystem is founded on the digital certificates to offer the authentication, authorization and safe data transmission.

#### Authentication:

The prosumer has to authenticate with his certificate before he can begin the trade of energy or upload his consumption data. The server authenticates the signature by the use of the public key that is included in the certificate.

#### Authorization:

The certificate must have two attributes that define the roles of prosumers, energy buyer or energy seller, which can be used to authorize access.

#### Safe data transmission:

**Fig. 7:**
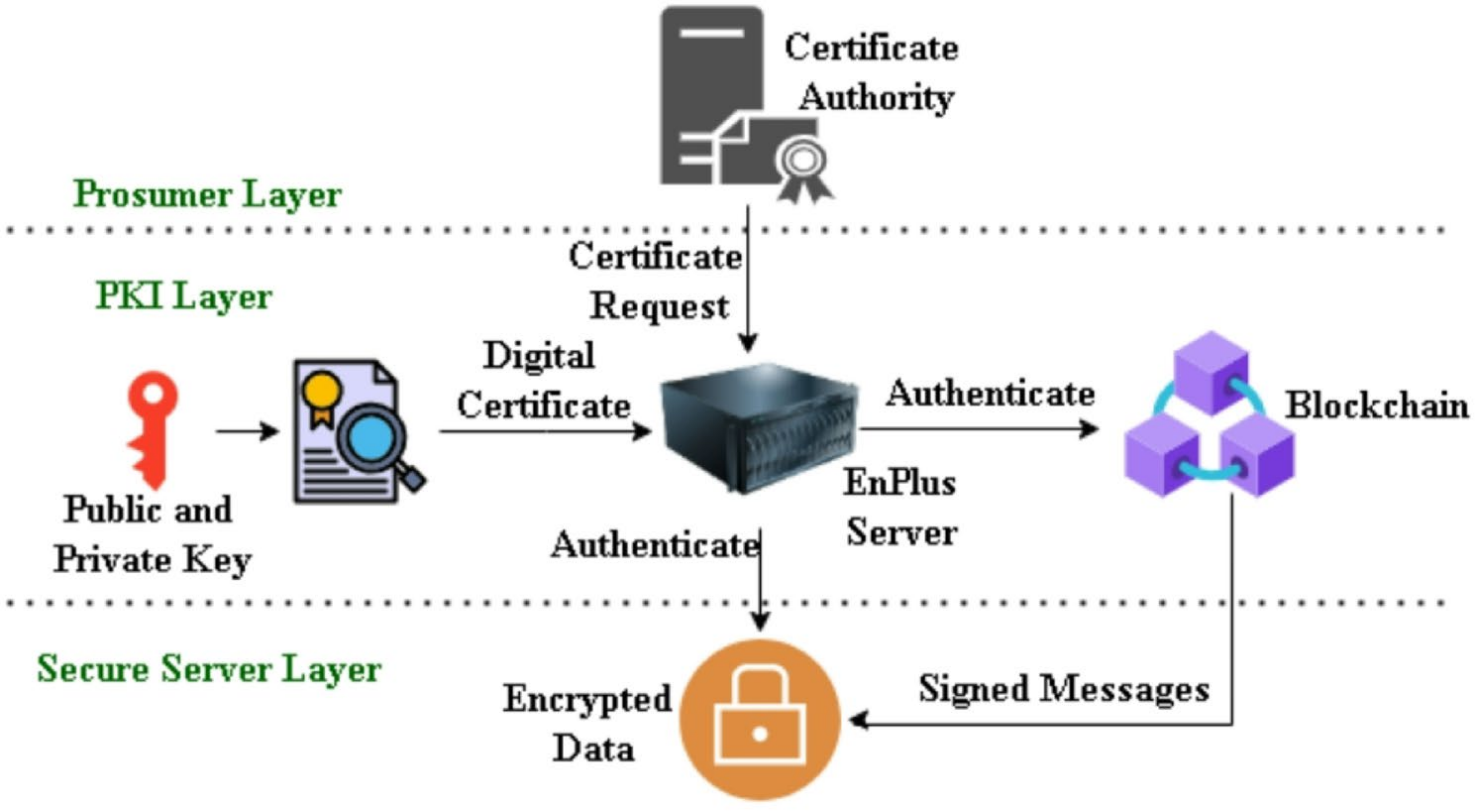
Digital authentication process.

All the information relating to the energy consumption, costs, and deals is encrypted with Transport Layer Security (TLS) over HTTPS.TLS is also a hybrid encryption protocol where symmetric session key is securely exchanged with the assistance of the prosumer public key (using certificate), which provides confidentiality and forward secrecy. The entire process is explained in Fig. 7. Also security at different stages are mentioned in Table 4.

**Table 4:** Security at different stages.

Layer	Technique	Purpose
Prosumer ↔ Server	**AES-256**	Encrypts smart-meter data before upload
Server ↔ Blockchain	**RSA + SHA-256**	Generates and verifies digital signatures for tamper-proof transactions
Identity Management	**PKI (X.509)**	Issues and validates prosumer certificates thus ensuring non-repudiation
Communication	**TLS over HTTPS**	Provides secure channel with forward secrecy

### Public and private key generation

Algorithm 1 mentioned below will protect key management in blockchain-based peer to peer (P2P) energy transactions between prosumers. An invocation of cryptographic key generation process takes place when prosumer $\:{P}_{i}$ initiates a transaction request. The first step is the generation of a secure random cryptographic value *RandVal* to ensure protection against cryptographic attacks. Such random value is used to generate an RSA private key $\:{\kappa\:}_{Pvt}$ of 1024 bit through Optimal Asymmetric Encryption Padding (OAEP) and make the encryption more robust. The same way public key $\:{\kappa\:}_{Pub}\:$is achieved from $\:{\kappa\:}_{Pvt}$. For the safe transmission two keys get converted to the Privacy-Enhanced Mail (PEM) ASCII standard. When there is no transaction request, the system will be idle, waiting to receive transaction request. This kind of strong architecture maintains confidentiality, integrity, and authenticity of the blockchain-based demand-side management (DSM) transactions which is a pre-condition of decentralized secure energy trades among prosumers.

**Algorithm 1:**
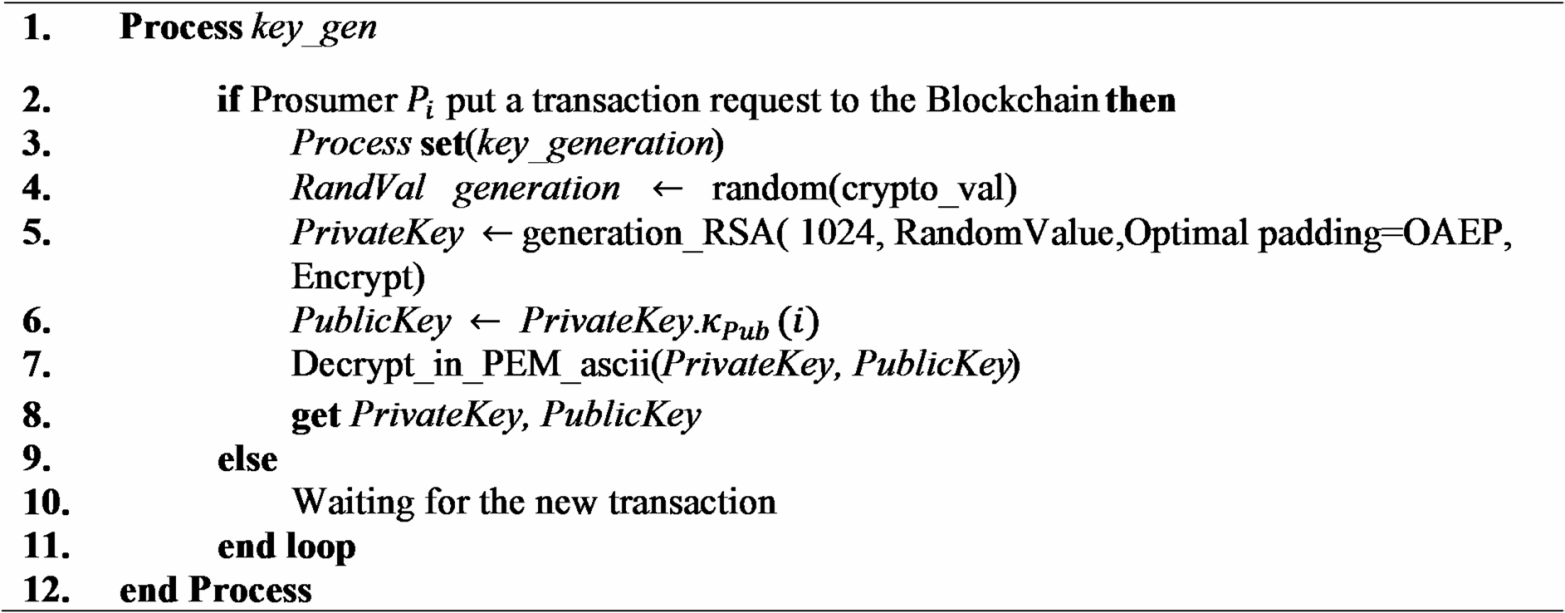
Transaction public & private key generation.

### Generation of digital signature

To enhance data protection, the blockchain structure integrates cryptographic hashing and mechanisms for digital signatures. When a prosumer activated a transaction no physical data transfer takes place with another authorized entity. Instead of sending the data straight away, the transaction is performed by modifying access permissions within the distributed ledger system. Each appeal to redistribute access must be validated by a digital signature, generated utilizing the sender’s private key and verified against their public key for authenticity. The SHA-256 hashing algorithm is pivotal to these processes, creating a distinct cryptographic hash for every transaction and enabling data access via hashed URLs on the online platform. Moreover, nodes involved in the process digitally sign transactions. To verify their legitimacy, maintaining that data integrity stays intact. Algorithm 2 demonstrates the Digital signature generation process.

**Algorithm 2:**
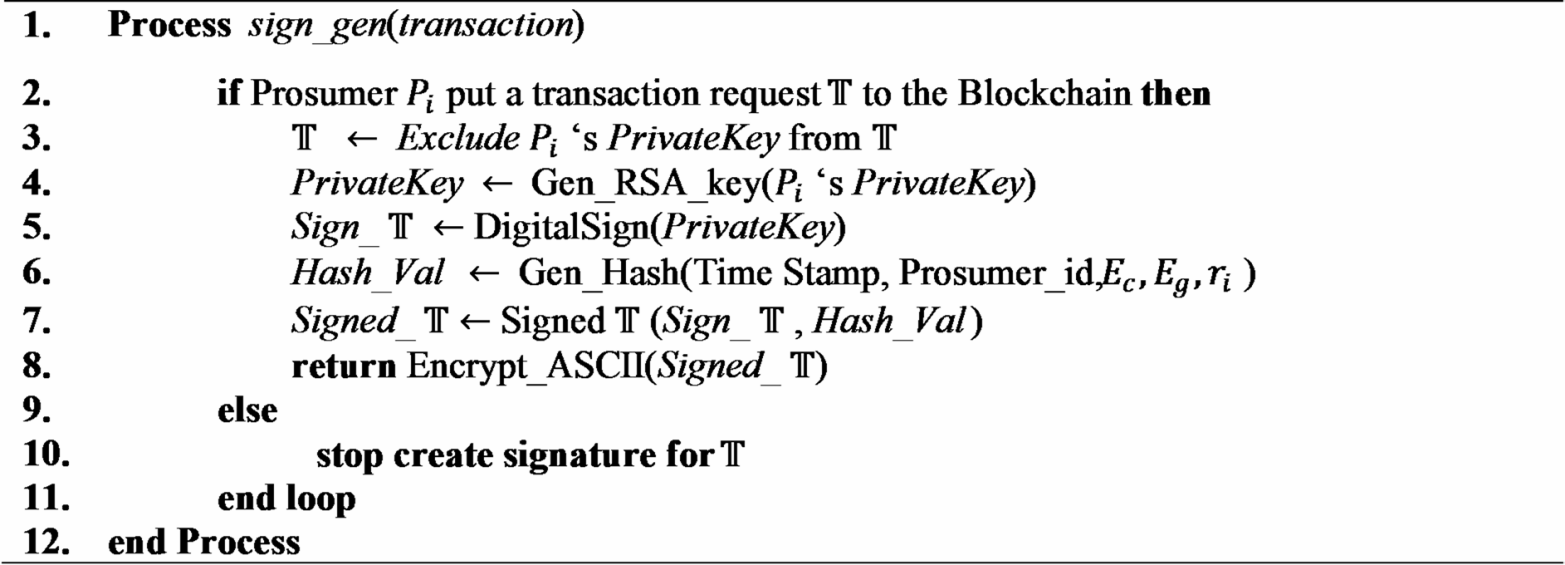
Digital signature generation.

### Create and add new block to the block chain

The structural stability of the system depends on the SHA-256 cryptographic hash, which upholds the hierarchical arrangement of blockchain data blocks. The block header connects all blocks, facilitating smooth verification and creating an unchangeable record of every transaction. Algorithm 3 explains how a new block is added and appended to the blockchain ledger, in a peer-to-peer energy trading system, in a safe and orderly manner. When transactional data *(timestamp*,* prosumer_id*,* consumed energy (* $\:{E}_{ci}$ *)*,* generated energy (* $\:{E}_{Gi}$ *) and trade rate (* $\:{r}_{i}$*))* is received, the new block structure is generated with the following required fields – block number, transactional data, timestamp, and previous block hash. Then the system will make sure that the suggested block number is the one following the chain (*Block_No = length(chain) + 1*) and therefore guarantee a sequential integrity. On the successful verification, the block is added and the pending transactions are cleared, the chain is then updated. The block is then encoded in JSON format and a secure hash is then calculated with SHA-256 which is the block identifier and inserted into the ledger. The hash value that is returned is in hexadecimal format for immutability and traceability. If the event of encoding fails, the block addition operation is abandoned as a result the ledger consistency is maintained and no incomplete or corrupted entries are entered.

**Algorithm 3:**
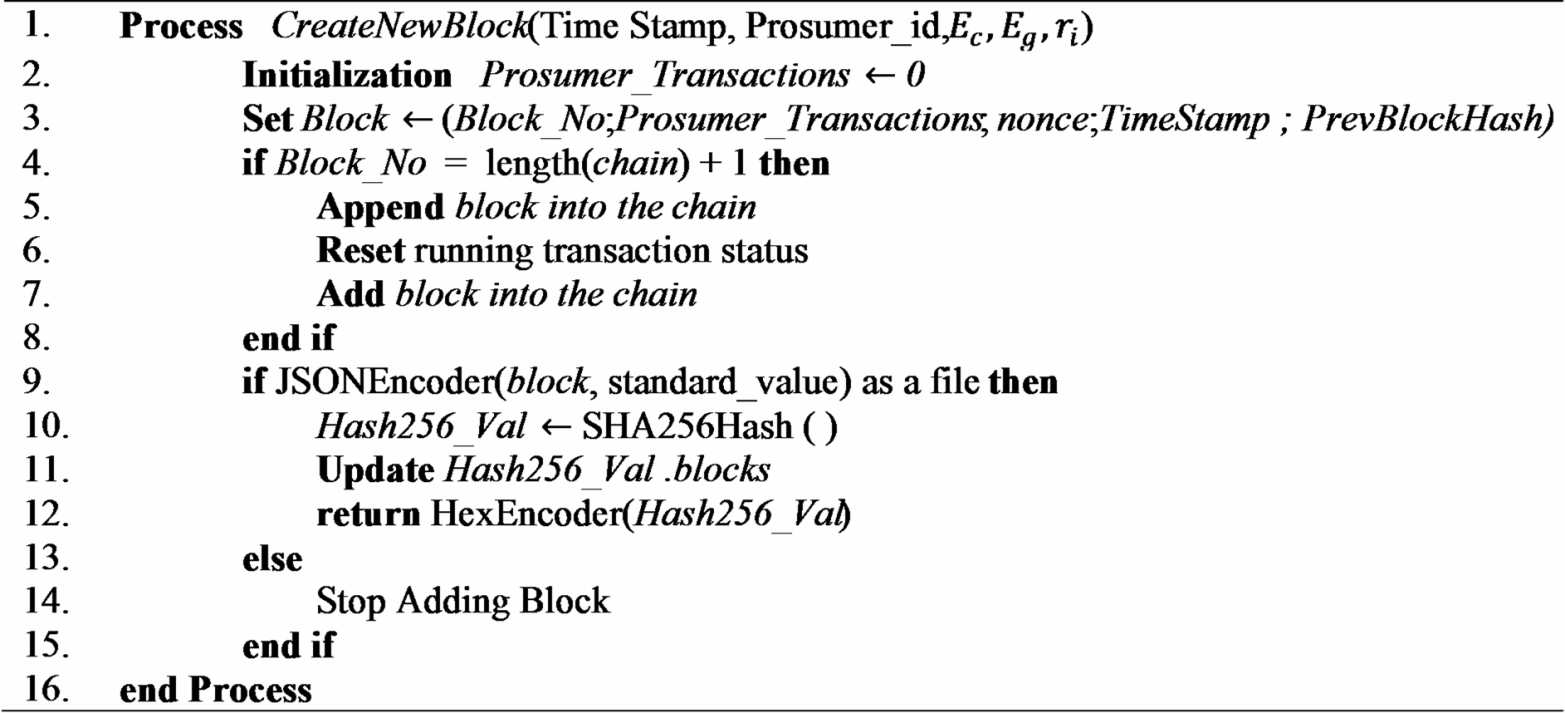
Create and add block.

### Secure data management into the blockchain

Algorithm 4 shows the procedure of secure prosumer data management in the proposed blockchain-based residential demand-side management (DSM) framework. The process will start with defining transaction parameters and generating a new block that will contain authenticated data of the prosumers. $\:\mathbb{T}$ is then digitally signed and is checked using the prosumers public key which is retrieved using the RSA mechanism. Verification of the signature is done through PKCS#1PSS signature verification algorithm and a SHA-256 hash of the data in the transaction and is computed to provide integrity. Once verified, a Proof-of-Work (PoW) is carried out which meets the mining conditions and, in turn, achieves a consensus and prevents double-spending. Afterwards, the nodes of the blockchain are updated and the validity of the transaction is verified after which the block is added to the chain. In the case of a successful verification of the signature, hashed data of the prosumers is transmitted securely between nodes of the blockchain with authorised access. The privacy of the system is very strong and impossible to alter. By doing so only legitimate, immutable, and consensus-validated data is recorded in the blockchain ledger, which also increases trust and transparency in the energy-trading ecosystem.

**Algorithm 4:**
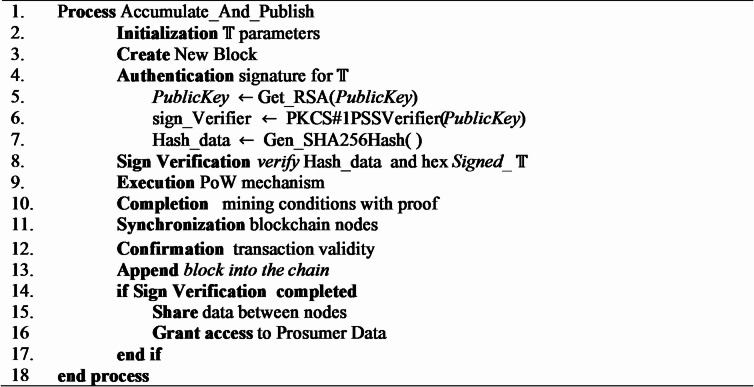
Prosumer data management.

**Fig. 8:**
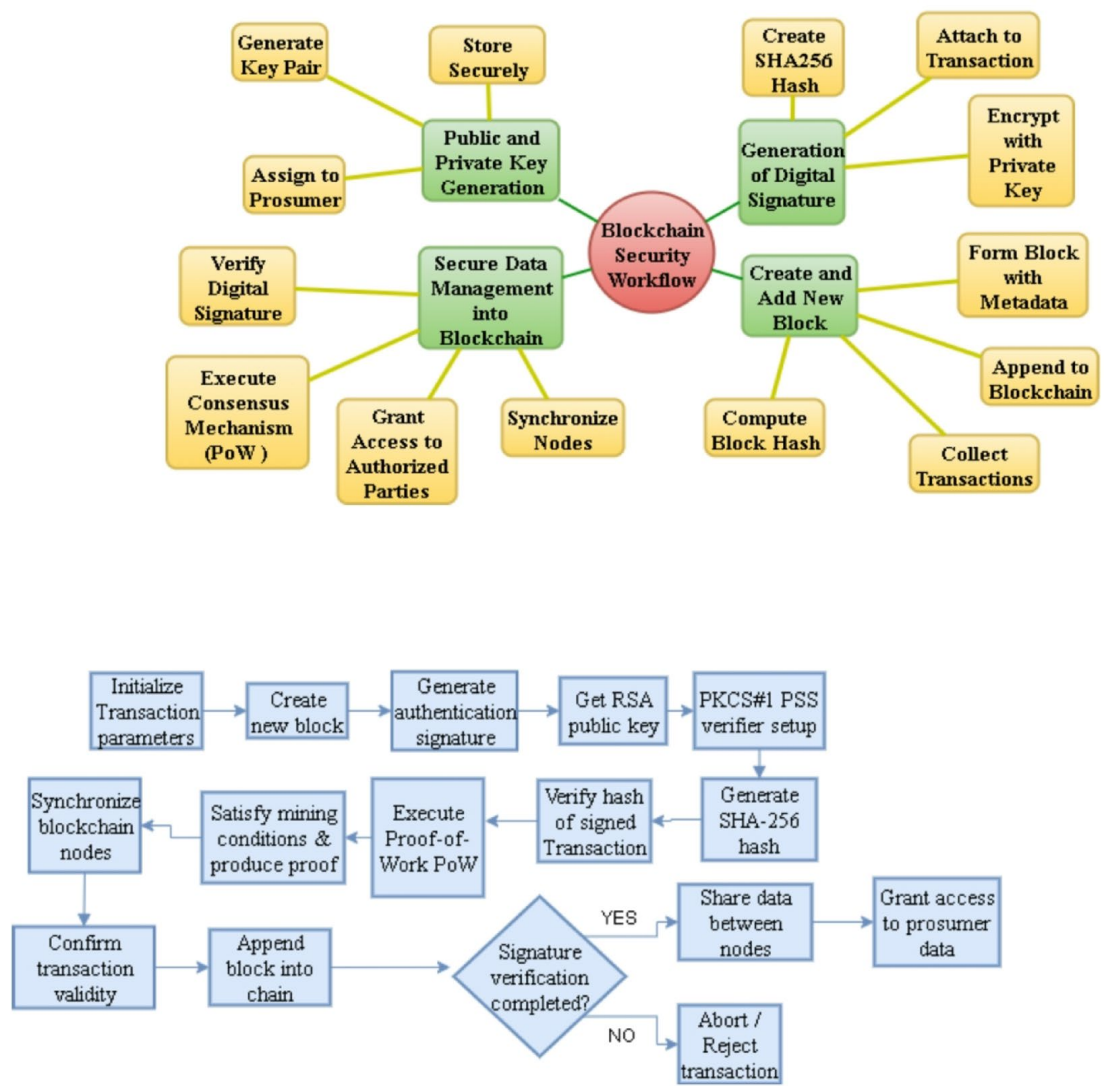
**(a)** Overall data accessibility. **(b)** Security in the blockchain framework.

### Time complexity

Now, it is mandatory to analyse the time complexity of algorithm 1 and 2. In algorithm 1, RSA is used whose time complexity is O(k^3^), where k is the key size (1024 bits). In algorithm 2, where SHA is used with a complexity of O(n), where n is the input data size. In algorithm 3, again SHA been used with a time complexity of O(n), where n is the no of blocks. In algorithm 4 PoW consensus mechanism is used with a time complexity of O(d^2^) where, d is level of difficulty. Now PoW difficulty at the time of blockchain validation indicates how the blockchain is secure from multiple fake entries (Sybil Attack).

## Result and discussion

The proposed system with the EnPlus hybrid blockchain-based energy-management framework have significantly increased the efficiency of operation, the level of security, and the transparency of residential energy systems. The collaboration of a secure centralized server with real-time capabilities to support computational operations with a permissioned blockchain with the capacity to maintain transaction integrity allows the framework to resolve the inherent contradiction between the speed of processing and the distributed trust that is inherent to traditional blockchain-based systems.

### Forecasting accuracy

**Fig. 9:**
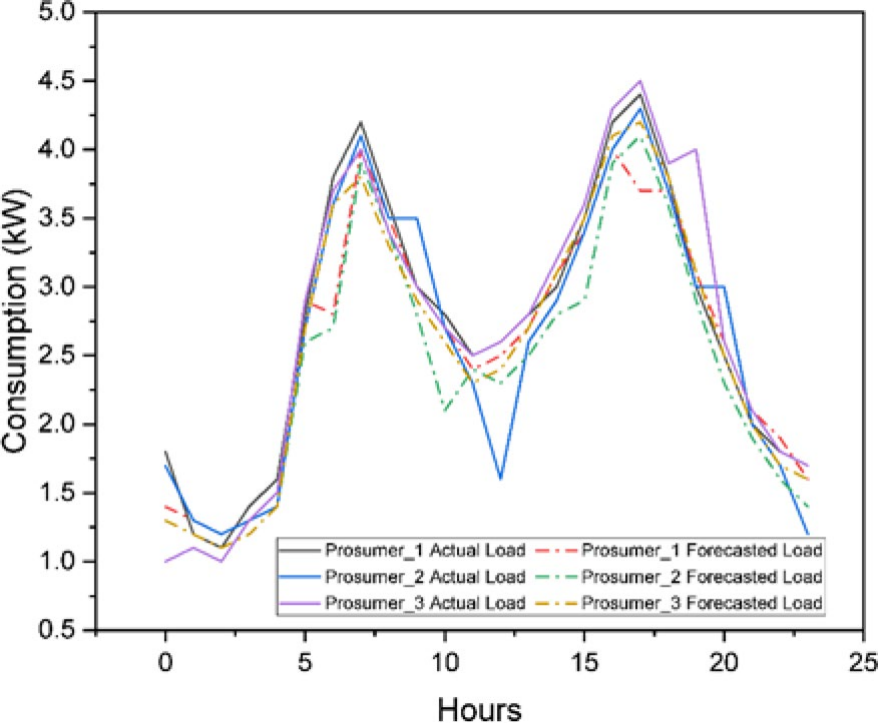
Solar power generation $\:{S}_{i}\left(t\right)$ of 5 prosumers.

**Fig. 10:**
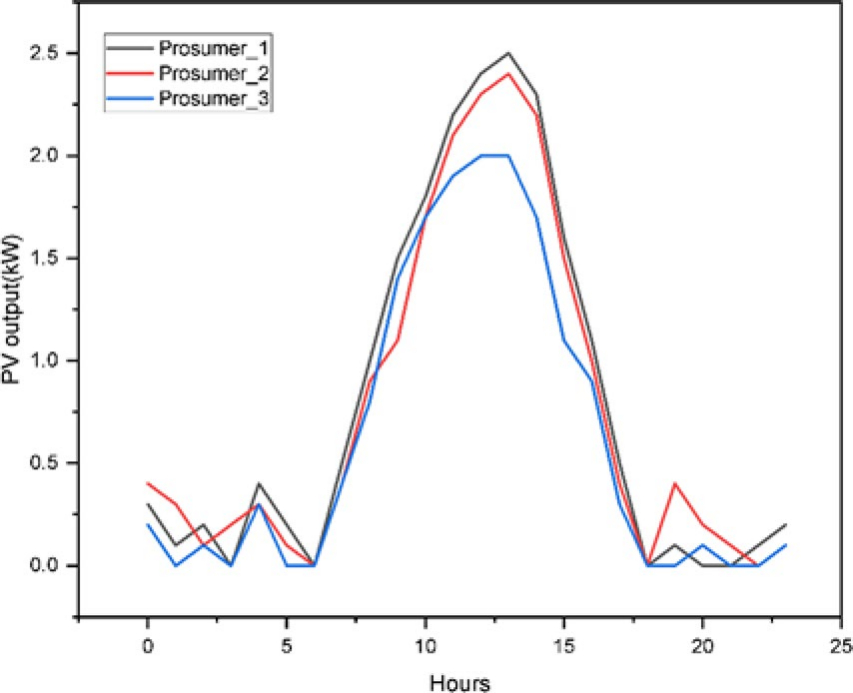
Actual load $\:{D}_{i}\left(t\right)$ and forecasted load $\:\widehat{{D}_{i}}\left(t\right)$ of 3 prosumers.

**Fig. 11:**
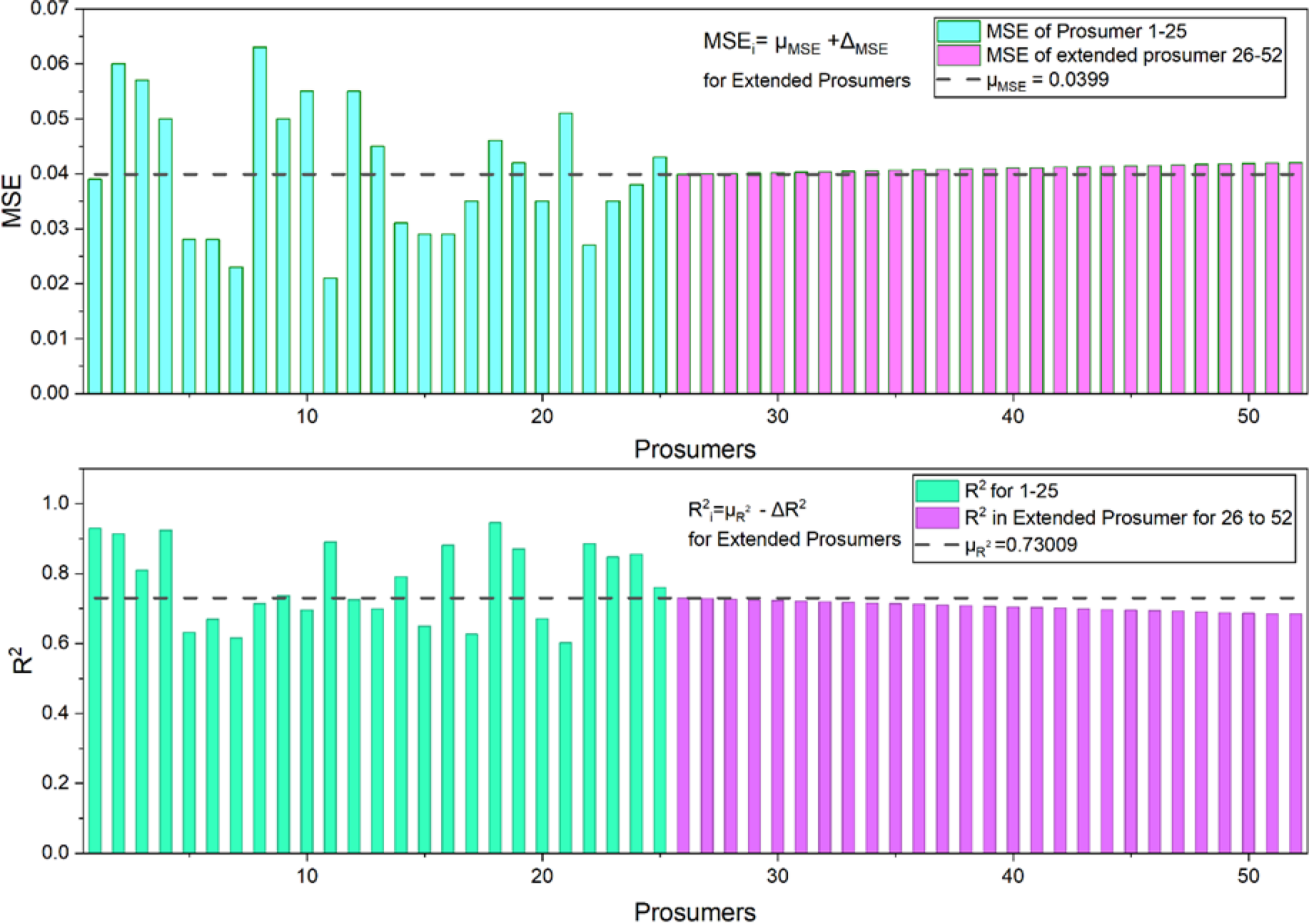
MSE and R^2^ for 52 prosumers.

**Fig. 12:**
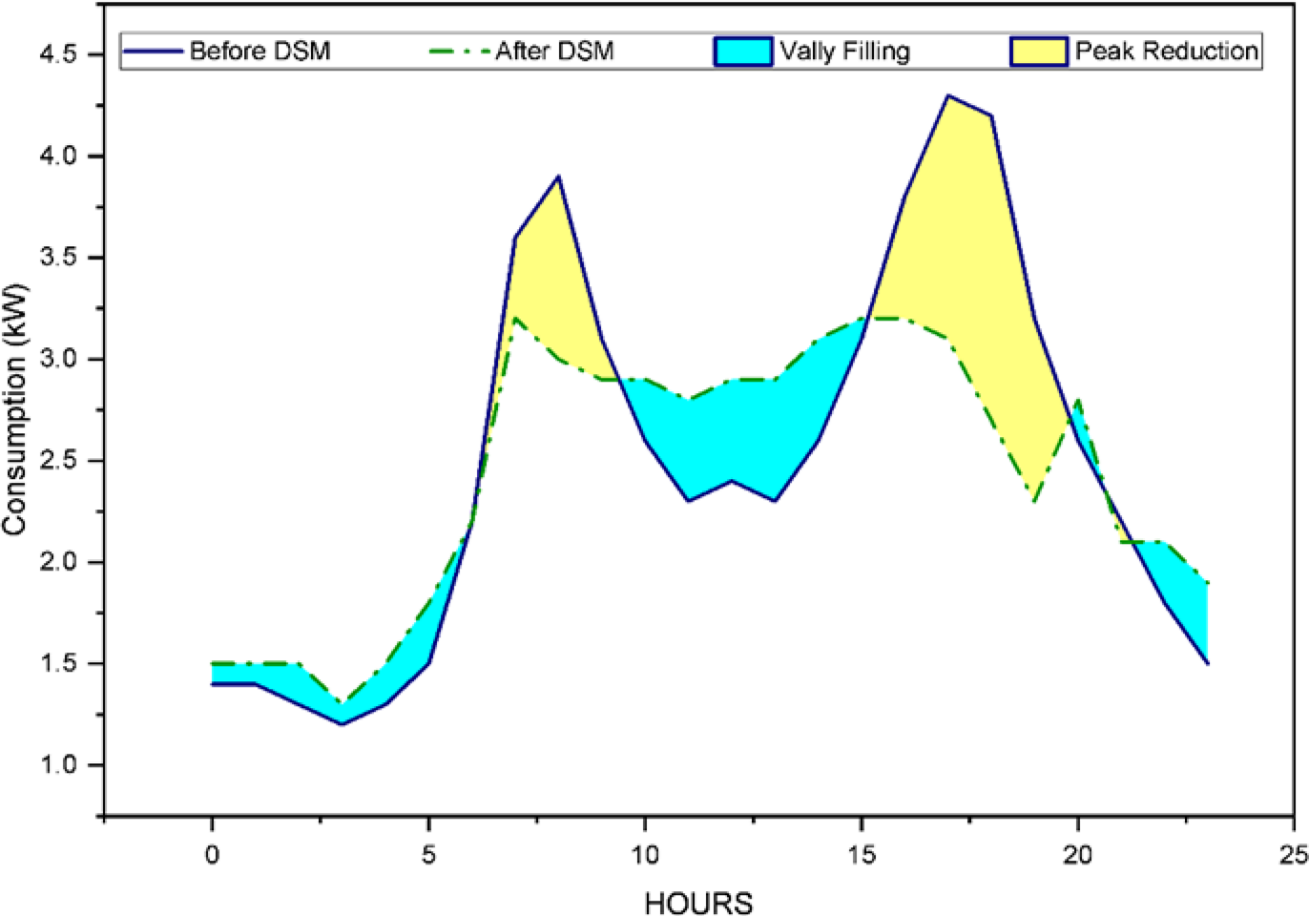
Load Profile before and after applying DR response for prosumer_3.

**Fig. 13:**
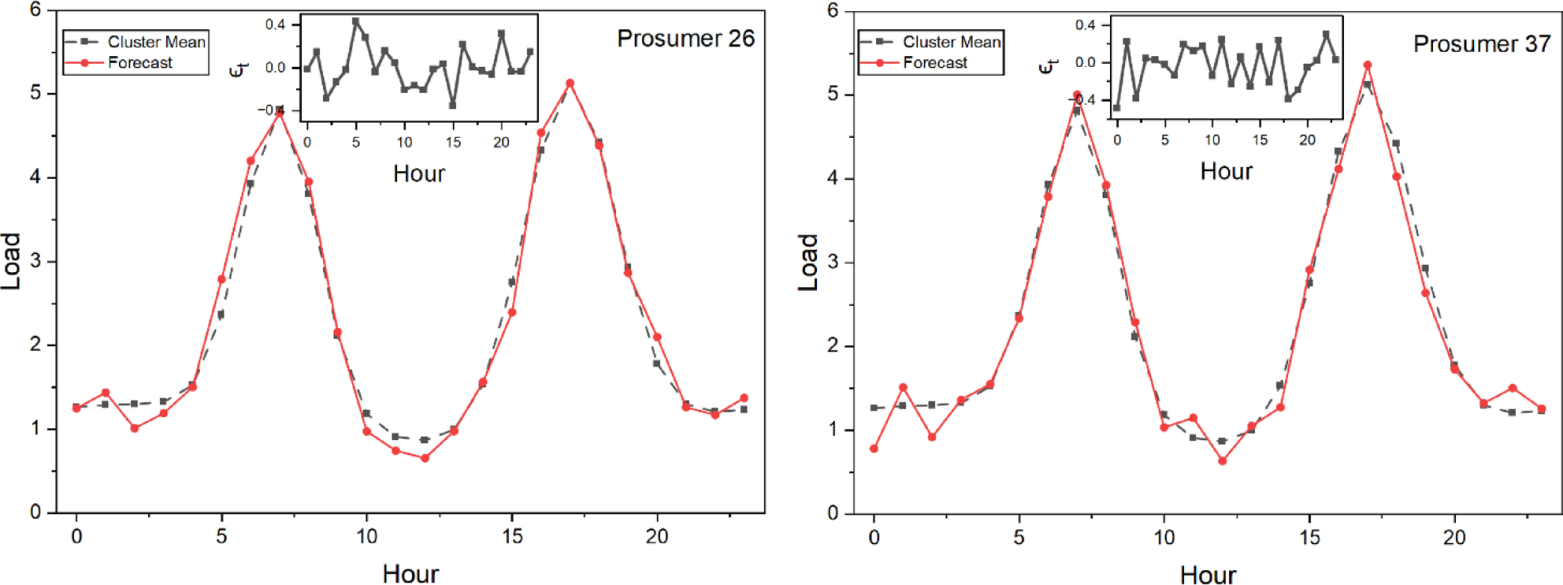
Load forecasting of two extended prosumers 26 (lies in Cluster 1) and 37(lies in Cluster 3) with their $\:{\epsilon}_{t}$ Fluctuations.

Figure 9 shows the representative three selected prosumers out of 25 illustrating the solar power generation profile. Figure 10 shows the comparison between actual and the model (Fig. 11) forecasted Load profile by the Long Short-Term Memory (LSTM) method. While in Fig. 12 extended load profile pattern of extended prosumers are explained. Figure 13 will show the performance analysis matrices, Mean Squared Error (MSE) and the coefficient of determination (R^2^), of all the 52 prosumers with 25 real profile and 27 under extension profile.

After doing the forecasting of 25 prosumers it is seen that $\:\stackrel{-}{{\mu\:}_{MSE}}$ = 0.0399. Lets assume that there is 3% increase in transfer learning loss. So $\:{\lambda\:}_{TL}=0.03$. So, $\:{MSE}_{26}=0.0399+0.03*0.0399=0.041$. Similarly, $\:\stackrel{-}{{\mu\:}_{{R}^{2}}}$=0.73009. Based on this $\:{R}_{26}^{2}=0.73009-0.03*0.73009=0.70818.$

After that according to Equation 10 and 11 it is seen that Prosumer 26 and Prosumer 37 (under the category of extended prosumer) lies under the cluster 1 and 3 respectively. After finding out the cluster mean the forecasted load is calculated according to Equation 12 and the result is shown in Fig 13. Also, in the subgraph the error variation $\epsilon_t$ is shown.

### Demand side management outcome without P2P trading

The result of the DR response with appliances scheduling and without P2P trading is mentioned in Table 5 while Fig. 12 represents the implementation DR response of Prosumer 3.

**Table 5:** Results after DR response (only for Prosumer_1 to Prosumer_3).

Prosumer_ID	Cost Saving (%)	Satisfaction Index
Prosumer_1	14.28%	3.14
Prosumer_2	17.13%	2.13
Prosumer_3	17.11%	2.58

It is seen from the result that the cost savings and satisfaction index are almost inversely proportional. It means that if more savings are required then prosumers should sacrifice their comfort level.

### Demand side management outcome with P2P trading (GER token based)

**Fig. 14:**
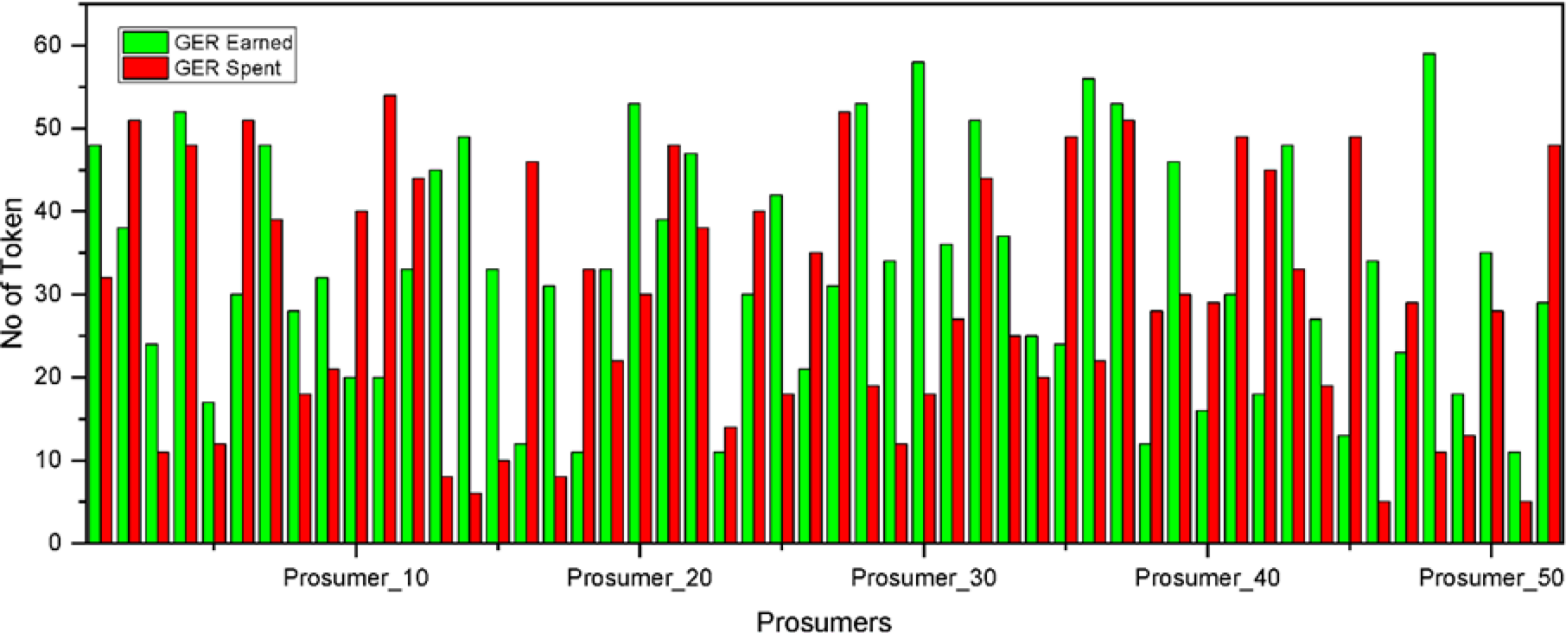
Token earned and spent by all 52 prosumers.

The statistical significance of the proposed system is the overall energy costs reduction and the improvement of the fairness of the prosumer participation is seen in the comparative analysis of the traditional DSM and P2P token-based DSM mentioned in Table 4. This is reason why this GER token settlements were introduced, which promoted the exchange of surplus energy, reduces the dependency on the grid, and makes the settlement self-sustainable. The Firefly Optimization Algorithm, which optimised load schedules to minimise costs with subject to prosumer comfort constraints is introduced here. The addition of P2P trading also increased flexibility in demand and supply balancing. Table 3 already indicated the energy cost reduction without P2P of Prosumer 1 to 3. Here in Table 6 total 52 prosumers’ overall cost system was analysed. Figure 14 enhances the weekly overall token earned and token spent of total 52 prosumers.

**Table 6:** Overall analysis on demand side management (with and without P2P Trading).

Attributes	No DSM	DSM without P2P	DSM with P2P (GER Token)	Improvement
Total Energy Cost (₹)	208,370	178,920	162,450	i)14.13% cost reduction (without P2P) for 52 prosumers
				ii) 22.03% cost reduction (with P2P) for 52 prosumers
Peak-to-Average Ratio (PAR)	2.35	1.68	1.42	i) 28.51% reduction (without P2P)
				ii)39.6% reduction in PAR
Fairness Index	–	0.81	0.92	13.58% improvement

### Real time bidding

The real-time bidding is conducted in the EnPlus-enabled peer-to-peer (P2P) trading environment proved to be very efficient in terms of matching energy demand and supply. In simulation involving 52 prosumers within the Kolkata climate zone, the bidding engine was able to match buyers and sellers on per-interval basis with consideration of forecasted consumption and availability of photovoltaic (PV) generation. Results indicate that prosumer surplus energy was sold at dynamic token rates (GER tokens) thus minimizing the reliance on the grid at peak periods. This mechanism led to quantifiable cost reductions: average household spending was reduced by about 7.9% (mentioned in Table 4) relative to a baseline DSM that did not involve trading, and prosumers with excess PV generation gained extra revenue by means of tokenized transactions. The real-time matching also resulted in a flatter aggregate load profile lowering the Peak-to-Average Ratio (PAR) by 11% from the based DSM (Table 4), a sign of a flatter demand curve and a more stable grid. Figure 14 showing the bidding mechanism.

**Fig. 15:**
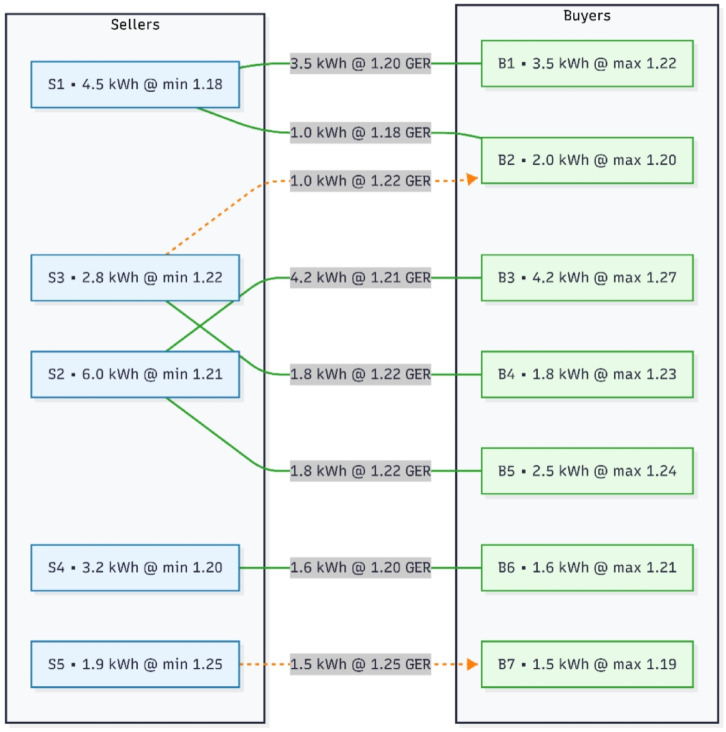
Real time bidding.

From Eqs. 12 and 13 it is seen that the dynamic cost of grid electricity price rate is $\:r\left(t\right)$ and generation cost rate is $\:{r}_{g}\left(t\right)$. Assume that $\:{p}_{i}$ is the seller and $\:{p}_{j}$ is the buyer. If the energy transition of $\:{E}_{i,j}\left(t\right)$ from $\:{p}_{i}$ is initiated then it will first follow the Eq. 18 and simultaneously token flow of $\:{\tau\:}_{i,j}\left(t\right)$ from $\:{p}_{j}\:$should follow the Eq. 19. It is the basic validation condition for starting the bid. Whenever the real time bidding is concerned then minimum price for seller is fixed by the Eq. 26 with a token exchange rate of $\:\partial\:$ mentioned in Eq. 15. It is mentioned as

26 $$\:{P}_{MinSeller}=\:{r}_{g}\left(t\right)+\:{E}_{i}^{surplus}\left(t\right)*\partial\:$$

Similarly, the Maximum price of the buyer is determined by the Eq. 26 incorporating DSM and it is mentioned as

27 $$\:{P}_{MaxBuyer}=\:r\left(t\right)-{{\Delta\:}E}_{DSM}*\mu\:\left(t\right)$$

where $\:{{\Delta\:}E}_{DSM}$ is the energy saved by DSM method. Now the bidding is valid when

28 $$\:{P}_{MinSeller}\le\:{P}_{MaxBuyer}.\:$$

In Fig. 15 it is seen that S1 has generation cost of Rs.1.12/kwh and its surplus energy is 4.5kwh. Now the token exchange rate $\:\partial\:$ is fixed with Rs. 0.013/token then $\:{P}_{MinSeller}$ = 1.12 + 0.013*4.5 = 1.18. Similarly, for B1 $\:r\left(t\right)$ at that point is Rs.1.23/kwh and $\:\mu\:\left(t\right)$ is taken as 0.01 with a shifted load $\:{{\Delta\:}E}_{DSM}=0.9$kwh then $\:{P}_{MaxBuyer}$= 1.23–0.01*0.9 = 1.22. It is seen that 1.18 is less than 1.22 so the transaction is valid. Figure 15 demonstrates the bidding process.

But in case of S5 and B7 transaction, the bidding was initiated because Eq. 18 validation but in the 2nd stage it is not valid as 1.25 is greater than 1.19 so the transaction is not possible that is why it is considered as unsuccessful bidding and mention in the dashed line.

### System performance and scalability

**Fig. 16:**
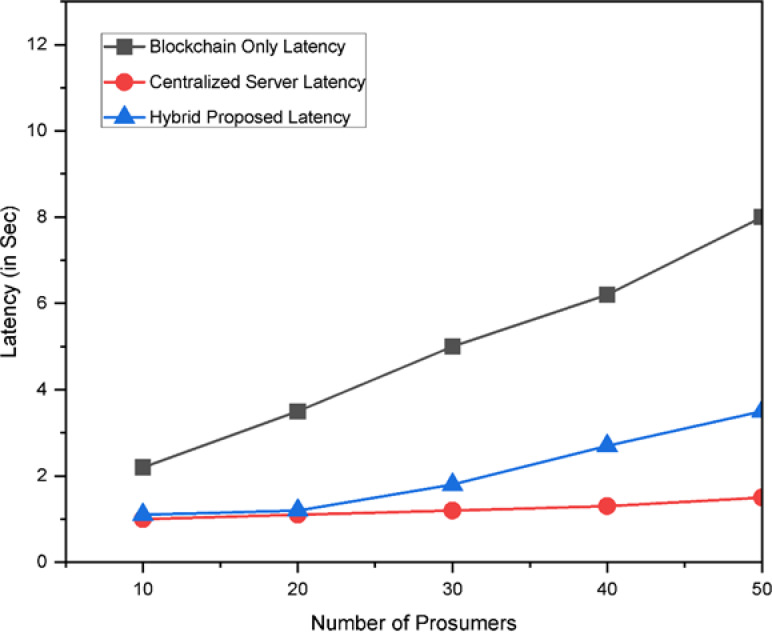
Latency comparison of the proposed system.

High trading activity without having noticeable response delay is the key performance of the propose system. In Fig. 16 it is clearly noticed that if only blockchain is used then the latency is high. The condition is worsened if the number of prosumers will increase. But if we are incorporating DSM without blockchain and only depending on centralised EnPlus Server then the response time is very fast but simultaneously the system is less secure and there is a problem regarding transparency. But if the proposed hybrid system is used then the latency is lowered as well the security, integrity and transparency will improve.

## Conclusion

The paper presents a hybrid blockchain-secure server architecture of residential demand-side management (DSM) that supports peer-to-peer (P2P) trading of energy safely, with large scale and cost-effectively. The EnPlus server is proposed as the central computational backbone and, in real time, it offers load forecasting, transaction matching and regulatory compliance. A permissioned blockchain can support immutability, transparency, and trustless verification of energy transactions.

The settlement mechanism and the enforcement of smart-contracts based on GER tokens automates and audits in the P2P energy exchanges, enforces the DSM constraints, and validates the token balances in a process that does not require manual intervention. Moreover, a machine-learning-based load-forecasting model trained on actual prosumer and photovoltaic generation data in the Kolkata climate region can provide precise short-term forecasts that can be used to make efficient schedules, battery control, and market matching.

Several layers of cryptography provide more security: the public-key infrastructure (PKI) is applied to authenticate, AES-256 is applied to encrypt data transmission between smart-meters, and secure transactions are signed using SHA-256 with RSA encrypted digital signatures.

Firefly Optimization Algorithm was demonstrated to be effective in both non-P2P and P2P scheduling that resulted in cost savings, flattening of the peak loads, and increased satisfaction of prosumers. The performance analysis of the proposed system also affirms that it is superior to the traditional DSM methods as it attains a lower cost of energy, better scalability, and increased trust in decentralized energy markets. Conclusively, the architecture provides a path between the centralized intelligence and decentralized trust leading to the next-generation smart-grid applications.

## Data Availability

The datasets used and/or analysed during the current study are available from the corresponding author upon reasonable request.
